# Musashi-2 Deficiency Triggers Colorectal Cancer Ferroptosis by Downregulating the MAPK Signaling Cascade to Inhibit HSPB1 Phosphorylation

**DOI:** 10.1186/s12575-023-00222-1

**Published:** 2023-12-01

**Authors:** Xiaole Meng, Xiao Peng, Wanxin Ouyang, Hui Li, Risi Na, Wenting Zhou, Xuting You, Yuhuan Li, Xin Pu, Ke Zhang, Junjie Xia, Jie Wang, Guohong Zhuang, Huamei Tang, Zhihai Peng

**Affiliations:** 1 Organ Transplantation Institute of Xiamen University, Fujian Provincial Key Laboratory of Organ and Tissue Regeneration, School of Medicine, Xiamen University, Xiamen, China; 2 National Institute for Data Science in Health and Medicine, Xiamen University, Xiamen, China; 3 Department of Pathology, Xiang’an Hospital of Xiamen University, School of Medicine, Xiamen University, Xiamen, China; 4 Department of General Surgery, Organ Transplantation Clinical Medical Center of Xiamen University, Xiang’an Hospital of Xiamen University, School of Medicine, Xiamen University, Xiamen, China

**Keywords:** Musashi-2 (MSI2), HSPB1, Ferroptosis, Colorectal Cancer (CRC)

## Abstract

**Background:**

Musashi-2 (MSI2) is a critical RNA-binding protein (RBP) whose ectopic expression drives the pathogenesis of various cancers. Accumulating evidence suggests that inducing ferroptosis of tumor cells can inhibit their malignant biological behavior as a promising therapeutic approach. However, it is unclear whether MSI2 regulates cell death in colorectal cancer (CRC), especially the underlying mechanisms and biological effects in CRC ferroptosis remain elusive.

**Methods:**

Experimental methods including qRT‒PCR, immunofluorescence, flow cytometry, western blot, co-immunoprecipitation, CCK-8, colony formation assay, in vitro cell transwell migration and invasion assays, in vivo xenograft tumor experiments, liver and lung CRC metastasis models, CAC mice models, transmission electron microscopy, immunohistochemistry, histopathology, 4D label-free proteomics sequencing, bioinformatic and database analysis were used in this study.

**Results:**

Here, we investigated that MSI2 was upregulated in CRC and positively correlated with ferroptosis inhibitor molecules. MSI2 deficiency suppressed CRC malignancy by inhibiting cell proliferation, viability, migration and invasion in vitro and in vivo; and MSI2 deficiency triggered CRC ferroptosis by changing the intracellular redox state (ROS levels and lipid peroxidation), erastin induced cell mortality and viability, iron homeostasis (intracellular total irons and ferrous irons), reduced glutathione (GSH) levels and mitochondrial injury. Mechanistically, through 4D-lable free proteomics analysis on SW620 stable cell lines, we demonstrated that MSI2 directly interacted with p-ERK and MSI2 knockdown downregulated the p-ERK/p38/MAPK axis signaling pathway, which further repressed MAPKAPK2 and HPSB1 phosphorylation, leading to decreased expression of PCNA and Ki67 and increased expression of ACSL4 in cancer cells. Furthermore, HSPB1 could rescue the phenotypes of MSI2 deficiency on CRC ferroptosis in vitro and in vivo.

**Conclusions:**

This study indicates that MSI2 deficiency suppresses the growth and survival of CRC cells and promotes ferroptosis by inactivating the MAPK signaling pathway to inhibit HSPB1 phosphorylation, which leads to downregulation of PCNA and Ki67 and upregulation of ACSL4 in cancer cells and subsequently induces redox imbalance, iron accumulation and mitochondrial shrinkage, ultimately triggering ferroptosis. Therefore, targeted inhibition of MSI2/MAPK/HSPB1 axis to promote ferroptosis might be a potential treatment strategy for CRC.

**Supplementary Information:**

The online version contains supplementary material available at 10.1186/s12575-023-00222-1.

## Introduction

Colorectal cancer (CRC), one of the most common human malignancies, accounts for approximately 10% of cancer mortality and incidence worldwide [1]. Despite recent progress in CRC cancer therapeutics, there are still some patients who may experience tumor recurrence or metastasis [2]. Therefore, there is an urgent need to identify effective therapeutic strategies and targets to improve the prognosis of CRC patients.

In the past decade, ferroptosis has emerged as a novel form of cell death that differs from autophagy, necroptosis, apoptosis, and other types and is characterized by aberrant intracellular iron accumulation, excessive lipid peroxidation and redox system imbalance [3]. Accumulating evidence has indicated that various molecules involved in ferroptosis play an important role in CRC cell death, growth inhibition and ROS accumulation [4–7]. Ferroptosis is closely associated with the weakened malignant phenotype of cancer cells, including proliferation, migration, invasion and metastasis [8–10]. Therefore, inducing ferroptosis might be an effective strategy for the treatment of cancers [11–13]. Furthermore, several anticancer agents, compounds and FDA-approved drugs have been proven to induce ferroptosis to resist tumor progression [14–17]. For instance, erastin, as an inducer of ferroptosis, functionally inhibits the cystine-glutamate transporter, resulting in the deprivation of antioxidant glutathione and ultimately oxidative cell death [14]. All these studies highlight that the induction of ferroptosis might be a promising therapeutic approach for CRC.

RNA-binding proteins (RBPs) are crucial for regulating various pathophysiological biological processes [18]. The RBP Musashi-2 (MSI2) has been identified as an oncoprotein in diverse tumors, including blood, breast, pancreatic, prostate, lung and colorectal cancers [19–21]. Notably, MSI2 has been implicated in cancer progression, particularly in the regulation of cell proliferation, metastasis, migration and invasion [21]. In addition, our previous studies identified other unique functions of MSI2 in metabolic reprogramming, immune regulation, intestinal microbial disorders and posttranslational modification [22–24]. More importantly, several RNA-binding proteins have recently been discovered to regulate ferroptosis in tumors, including glioblastoma [25], hepatocellular carcinoma [26, 27], head and neck cancer [28] and lung cancer [29]. Furthermore, targeting MSI2 with small molecule inhibitors has achieved beneficial effects in the treatment of CRC and acute myeloid leukemia [30, 31]. However, no study to date has explored the effects of MSI2 on ferroptosis, especially in CRC. Thus, the potential mechanism by which the RBP MSI2 regulates CRC ferroptosis deserves further investigation.

Heat shock proteins (HSPs) are the most abundant molecular chaperone proteins; their aberrant expression is usually caused by stress, such as heat shock, and they play a vital role in maintaining the proper folding and assembly of proteins [32, 33]. Recently, a variety of heat shock protein family members have been implicated in inhibiting ferroptosis in different disorders and cancers [33]. Among them, HSPB1, also known as HSP27, a small heat shock protein, has been validated to inhibit ferroptosis cell death in HeLa cells, glioblastoma, esophageal squamous carcinoma and bladder cancer cells [34–38]. In addition, HSPA5 has been reported to negatively regulate ferroptosis in pancreatic ductal adenocarcinoma (PDAC) and osteoarthritis by directly binding with the GPX4 protein [13, 39]. On the one hand, HSPB1 overexpression is closely associated with poor outcomes in multiple human cancers as it promotes cancer cell proliferation and metastasis and inhibits cell death [40–42]. On the other hand, activation of the mitogen-activated protein kinase (MAPK) signaling pathway is essential for sustaining cell viability and survival, and p38-MAPK activation further leads to an increase in HSPB1 expression levels and phosphorylation [38, 43–45]. The HSPB1 protein serves as a downstream substrate for MAPKAPK2 kinase, and MAPKAPK2 can further promote HSPB1 phosphorylation in response to stress, thereby effectively protecting against oxidative stress injury [46–49]. Although the role of HSPB1 in ferroptosis has been reported in several studies, the underlying molecular mechanism by which the RBP MSI2 regulates HSPB1 remains largely unknown.

In the present study, we investigated that MSI2 deficiency could inhibit the growth and metastasis of CRC cells in vitro and in vivo. Through proteomic analysis, we identified that MSI2 knockdown triggered CRC ferroptosis cell death by inactivating the p-ERK/p38/MAPK signaling pathway to inhibit HSPB1 phosphorylation, thus leading to intracellular redox imbalance, mitochondrial injury and iron accumulation. In addition, activation of HSPB1 attenuated the effects of MSI2 deficiency on CRC ferroptosis in vitro and in vivo. Thus, targeted inhibition of MSI2 to promote ferroptosis might be a potential treatment strategy for CRC.

## Materials and Methods

### Clinical Specimens

In this study, CRC tumor tissues and matched normal adjacent tissues were collected from the Department of Pathology, Xiang’an Hospital of Xiamen University. This study was approved by The Ethics Committee of Xiang’an Hospital of Xiamen University (Approval number: 20200722YJ001).

### Establishment of Stable Cell Lines

Plasmids were transfected using Transfect EZ 3000 plus (RD-SC2021-1). The control, MSI2 knockdown and overexpression plasmids in recombinant lentiviral vectors were synthesized by GeneChem (Shanghai, China). The MSI2 shRNA sequence was 5′-GTGGAAGATGTAAAGCAATAT‐3′. The pCDNA3.1 vector and HSPB1 Myc tag plasmids were constructed by Tsingke Biotechnology Co., Ltd. (Beijing, China). After purification of a large number of plasmids to yield endotoxin-free plasmids, stable cell lines were constructed by using lentiviral infection technology. The viral supernatants were collected 48 to 72 h after transfection and used to infect CRC cell lines; successfully transfected cells were then selected with 2 µg/mL puromycin. The transduction efficiency was measured by qRT‒PCR and Western blotting.

### Cell Culture, Reagents and Detection

The CRC cell lines LOVO, SW620 and HT29 were purchased from the Type Culture Collection of the Chinese Academy of Sciences (Shanghai, China) with STR certification. The cell lines were cultured in Dulbecco’s modified Eagle’s medium (HyClone) supplemented with 10% fetal bovine serum (Gibco) and 1% penicillin/streptomycin (HyClone). Cell viability was determined by Cell Counting Kit-8 (CCK-8) assay (Yeasen, #40203ES76) according to the manufacturer’s instructions. The colony formation assay was performed to assess the proliferation of cells. Briefly, stable CRC cells were resuspended and seeded into 6-well plates at a density of 1000 cells per well, cultured for 14 days at 37 °C in a humidified incubator, fixed with 4% paraformaldehyde and stained with 0.1% crystal violet to count the clones. In vitro cell Transwell migration and invasion assays were conducted according to our previous report [36]. The membrane of each transwell was stained, cut and spread on a microscope slide, and the cells were counted in 4 random fields visualized at 10× magnification. For induction and inhibition of ferroptosis, stable CRC cells were treated with erastin (Selleck, #S7242) or ferrostatin-1 (Selleck, #S7243) for 8–24 h, and then the corresponding phenotypes were examined. The levels of intracellular total iron, ferrous ions and reduced GSH were determined using the Cell Total Iron Colorimetric Assay Kit (Elabscience, #E-BC-K880-M), Cell Ferrous Iron Colorimetric Assay Kit (Elabscience, #E-BC-K881-M) and Reduced Glutathione (GSH) Assay Kit (Nanjing Jiancheng Bioengineering, #A006-2-1) according to the manufacturer’s instructions. All cultured cells were maintained in an incubator at 37 °C in a humidified atmosphere containing 5% CO_2_.

### RNA Extraction and Quantitative Real-time PCR (qRT‒PCR)

Total RNA from human CRC tissues or cells was extracted using TRIzol reagent (Invitrogen, # 15,596,026) according to the manufacturer’s instructions. cDNA was synthesized by using Fast-King gDNA Dispelling RT Super Mix (TIANGEN, #KR118). The PCR process was performed using a real-time fluorescence quantitative PCR instrument (Roche, LightCycler480 II, Germany) and Super Real Pre-Mix Plus (SYBR Green) (TIANGEN, #FP205) for quantification. Relative mRNA expression levels were calculated by the 2^−△△Ct^ method, and the human β-actin gene was used as an endogenous control for normalization. All primer sequences used were synthesized by Tsingke Biotechnology Co., Ltd. as listed below: *β-actin*: F: AAGGTGACAGCAGTCGGTT, R: TGTGTGGACTTGGGAGAGG; *MSI2*: F: ATCCCACTACGAAACGCTCC, R: GGGGTCAATCGTCTTGGAATC; *HSPB1*: F: TGGACCCCACCCAAGTTTC, R: CGGCAGTCTCATCGGATTTT; *ACSL4*: F: AGGACATTTAAAAACGCTATGGCA, R: GTCCCAAGGCTGTCCTTCTT; *FTH1*: F: TGAAGCTGCAGAACCAACGAGG, R: GCACACTCCATTGCATTCAGCC; *GPX4*: F: GAGGCAAGACCGAAGTAAACTAC, R: CCGAACTGGTTACACGGGAA.

### Western Blot Analysis

Western blot assays were performed as described previously [36]. Briefly, cell lysates were prepared in cold RIPA buffer (Solarbio, #R0010) containing InStab™ Protease Inhibitor Cocktail (EDTA-free) (Yeasen, #20124ES03) and InStab™ Phosphatase Inhibitor Cocktail (Yeasen, #20109ES05). The cell lysates were centrifuged at 15,000 × g for 15 min at 4 °C, and cell protein amounts were quantified with a Pierce BCA protein assay kit (Thermo Fisher, #23,227). Western blot assays were performed using standard techniques with the following antibodies in this study: MSI2 (Abcam, #ab76148), HSPB1 (PTMBIO, #PTM-6188), P-HSPB1(Ser78) (PTMBIO, #PTM-6652), TFRC (Affinity, #AF5343), FTH1 (Affinity, #DF6278), ACSL4 (PTMBIO, #PTM-6141), GPX4 (Abcam, #ab125066), actin (Sungene Biotech, #KM9001T), β-tubulin (Sungene Biotech, #KM9003T), P42/44 (CST, #4695S), P-P42/44 (CST, #4370S), P38 (ZENBIO, #200,782), P-P38 (ZENBIO, #310,091), MAPKAPK2 (ZENBIO, #R27028), P-MAPKAPK2 (ZENBIO, #310,236), MAPK13 (ZENBIO, #310,008), PCNA (ZENBIO, #200947-2E1), HRP Conjugated Goat anti-Rabbit IgG (HUABIO, #HA1001), and HRP Conjugated Goat anti-Mouse IgG (HUABIO, #HA1006). And grayscale analysis of western blotting results was performed using Image-J software.

### Co-Immunoprecipitation (Co-IP)

For the immunoprecipitation assay, the supernatants of SW620, LOVO and HEK293T cell lysates were subjected to immunoprecipitation with 2 µg of MSI2 (Abcam, #ab76148) and control IgG antibody for 8 h at 4 °C, and 50 µl of Protein A/G IP magnetic beads (Epizyme, Cat. YJ201) were added to the cell lysates and incubated for 6 h at 4 °C. The immunocomplex beads were then washed five times with IP buffer containing Protease Inhibitor Cocktail and PMSF. Finally, the input cell lysates, flow-through buffer and immunocomplex beads were resuspended in 2× SDS loading buffer, boiled and examined by Western blotting.

### Flow Cytometric Analysis

Flow cytometric analysis was performed to examine the mitochondrial membrane potential (MMP), the levels of ROS and lipid ROS, and the percentage of dead cells. Briefly, SW620, LOVO and HT29 stable cell lines were seeded in 6-well plates for 24 h, washed twice with 1 × PBS, and then incubated with reagents from the JC-1 Mitochondrial Membrane Potential Assay Kit (LABLEAD, #J22202), CellROX™ Deep Red (H2DCFDA) (Invitrogen, #C10422), BODIPY™ 581/591 C11 (C11-BODIPY) (Invitrogen, #D3861) and Annexin V-APC Cell Death Detection Kit (KeyGEN BioTECH, #KGA1021) according to the manufacturer’s instructions. Finally, the stained cells were washed twice with 1 × PBS and then analyzed on a Beckman CytoFlex flow cytometer (Beckman, USA). The positive cell percentages and mean fluorescence intensity were calculated by using FlowJo v10 software.

### Immunofluorescence (IFC) Assay

IFC assays were performed as described previously [50]. Briefly, for CRC tissues, the sections were deparaffinized and subjected to antigen retrieval by Tris-EDTA buffer (pH 9.0) at 95 °C for 40 min; then they were blocked with 10% goat serum for 60 min at room temperature. Stable cells were seeded on coverslips in 24-well plates, fixed with 4% paraformaldehyde for 15 min after two washes with 1× PBS at room temperature, permeabilized in 0.5% Triton X-100 for 10 min after five washes with 1 × PBS, and then incubated with blockade buffer containing 5% BSA. Afterward, sections and cell coverslips were incubated with primary antibodies (MSI2 (Abcam, #ab76148), p-HSPB1 (Ser78) (PTMBIO, #PTM-6652), P-P42/44 (CST, #4370S)) for 8 h at 4 °C or incubated with reagents from the TUNEL Cell Death Detection Kit (FITC) (Yeasen, #40306ES50) and JC-1 Mitochondrial Membrane Potential Assay Kit (LABLEAD, #J22202) according to the manufacturer’s instructions. After five washes with PBST, the sections and cell coverslips were incubated with Alexa Fluor® secondary antibodies (Goat anti-Rabbit IgG H&L Alexa Fluor 594 (ZENBIO, #550,043), goat anti-Rabbit IgG H&L Alexa Fluor 488 (ZENBIO, #550,037)) and DAPI nuclei stain (ZETALIFE). Coverslips and sections were washed five times, and images were acquired using a Nikon confocal microscope.

### 4D Label-free Proteomic Analysis

The 4D label-free proteomics analyses were carried out by PTM Biolab (PTM Bio; Hangzhou, China). The differentially expressed proteins identified by proteomics analysis of stable SW620 cells are reported in the Supplementary materials. The functions of the genes corresponding to the differentially expressed proteins were analyzed and annotated by Gene Ontology (GO) analysis, Kyoto Encyclopedia of Genes and Genomes (KEGG) analysis, subcellular localization analysis, Clusters of Orthologous Groups of proteins (COG/KOG) analysis and gene set enrichment analysis (GSEA). The GO enrichment annotations included categories of biological processes, cellular components and molecular functions. KEGG pathway enrichment analysis employed the online service tool KAAS to annotate the protein’s KEGG database description. For subcellular localization prediction, we used Wolfpsort, an updated version of PSORT/PSORT II for the prediction of eukaryotic sequences. COG/KOG enrichment analysis was performed to identify significant clusters based on the KOG database (http://www.ncbi.nlm.nih.gov/COG/KOG). GSEA was conducted with the GSEA tool (http://www.broadinstitute.org/gsea). A two-tailed Fisher’s exact test was employed to assess the enrichment of the differentially expressed proteins against all identified proteins, and a corrected *p* value < 0.05 was considered to indicate significance.

### In Vivo Experiments

For xenograft tumor experiments, LOVO, HT29 and SW620 stable cells (1 × 10^6^ cells) were injected subcutaneously into female M-NSG mice (Shanghai Model Organisms Center, NOD.Cg-*Prkdc*^scid^*Il2rg*^em1Smoc^); 4–6 mice were used in each group, and the xenograft tumor volume was monitored every other day and calculated as volume = (length × width^2^)/2. The tumor weight was measured on Day 24 after the mice were sacrificed. For the metastasis model experiment, LOVO and SW620 stable cells (1 × 10^6^ cells) were injected into the spleens and tail veins of female M-NSG mice to establish liver and lung CRC metastasis models, and 5 mice were used per group. At the end of the 6-week experiment, the metastases were counted, and the liver and lung tissues were collected for histology. For colitis-associated colon cancer (CAC) induction, MSI2 knockout (*MSI2*^*−/−*^) transgenic and wild-type (*MSI2*^*+/+*^) C57BL/6 mice were generated by Shanghai Model Organisms Center, and the mice were treated with the carcinogen azoxymethane (AOM) (10 mg/kg) (Sigma) intraperitoneally and repeated 3% dextran sodium sulfate (DSS) (MP Biomedicals, mol wt. 36–50 kDa) three times by feeding. All mice were maintained and bred at the pathogen-free facility of Xiamen University Laboratory Animal Center. All experimental procedures were performed in accordance with the Guide for the Care and Use of Laboratory Animals approved by the Xiamen University Laboratory Animal Center (Approval number: XMULAC20190113).

### Transmission Electron Microscopy

The SW620, LOVO and HT29 stable cells (1 × 10^7^ cells) were harvested in tubes and fixed with 2.5% glutaraldehyde at room temperature in the dark for 2 h. The cell samples were then embedded in resin for ultrathin sections using a Leica EM UC7. The electron microscopy images were taken using a HITACHI HT7800 TEM 120 kV (Tokyo). Intact or shrunken mitochondria were counted in at least three different fields, and the percentage of shrunken mitochondria was calculated based on the total number of mitochondria in each field.

### Hematoxylin-eosin (H&E) Staining

In brief, fresh CRC or xenograft tumors and metastatic tissues were fixed with 4% paraformaldehyde at 4 °C overnight, paraffin embedded and sectioned. The sections were stained with hematoxylin and eosin and then dehydrated in an alcohol gradient and xylene. Histological H&E images were observed and photographed using a Leica Aperio Versa 200 microscope.

### Immunohistochemical (IHC) Staining

We performed immunohistochemical staining as previously described [22]. Briefly, IHC was carried out on paraffin-embedded CRC or in vivo model tumor tissues. The sections were then subjected to gradient hydration and heat-induced antigen retrieval and incubated with MSI2 (Abcam, #ab76148), HSPB1 (PTMBIO, #PTM-6188), P-HSPB1(Ser78) (PTMBIO, #PTM-6652), ACSL4 (PTMBIO, #PTM-6141), P-P38 (ZENBIO, #310,091), PCNA (ZENBIO, #200947-2E1) and Ki67 (Servicebio, #GB111499) antibodies at 4 °C overnight. Immunohistochemical staining was visualized with diaminobenzidine (DAB) techniques. The IHC images were scanned and captured with a Leica Aperio Versa 200 microscope, and the integrated optical density per area (IOD/area) of each IHC image was analyzed by Image-Pro Plus software.

### Bioinformatic and Database Analysis

The GEPIA2 (http://gepia2.cancer-pku.cn/) CRC databases were used to determine the transcriptional expression of MSI2 in normal and tumor tissues, and the GEPIA2 CRC and TIMER (http://https://cistrome.shinyapps.io/timer/) COAD databases were used to determine the *Spearman* correlations between MSI2 and ferroptosis-related genes. The UALCAN CPTAC (http://ualcan.path.uab.edu/) database was employed to analyze the protein expression of MSI2 in primary CRC and normal tissues. Online-available GSE14333 and GSE17536 datasets were downloaded from the Gene Expression Omnibus (GEO, https://www.ncbi.nlm.nih.gov/geo/) to determine the survival curves of MSI2 and/or HSPB1 by using the Kaplan‒Meier method with the log-rank test and the *Spearman* correlations between MSI2 and apoptosis-related genes. GDC TCGA datasets were downloaded from The Cancer Genome Atlas (TCGA, https://portal.gdc.cancer.gov/) to analyze the differential ferroptosis-related genes and KEGG pathway enrichment analysis in MSI2-high and MSI2-low patients. *Spearman* or *Pearson* correlation coefficients were used to determine the correlation between MSI2, HSPB1 and ferroptosis-related genes and pathway score in the TCGA database. The gene effect scores were determined by DepMap and DEMETER2 correlation analysis from The Cancer Cell Line Encyclopedia (CCLE, https://portals.broadinstitute.org/ccle) CRC cell line database. Ferroptosis driver and suppressor genes set were obtained from FerrDb (http://www.zhounan.org/ferrdb/current/). The PPI networks of HSPB1 and MAPK family genes were collected from STRING (https://string-db.org/).

### Statistical Analysis

The experimental data were analyzed using GraphPad Prism 7.5 software. The two-tailed unpaired or paired Student’s t test and Wilcox test were used for comparisons between two groups. One-way ANOVA and the Kruskal‒Wallis test were used to compare differences between more than two groups. All quantitative data are presented as the mean ± standard deviation of three independent experiments. A p value of < 0.05 was considered to indicate a statistically significant difference. *p < 0.05; **p < 0.01; *** *p* < 0.001; **** *p* < 0.0001; ns: not significant.

## Results

### MSI2 Expression is Upregulated in CRC and Positively Correlated with Ferroptosis Inhibitor Molecules

**Fig. 1:**
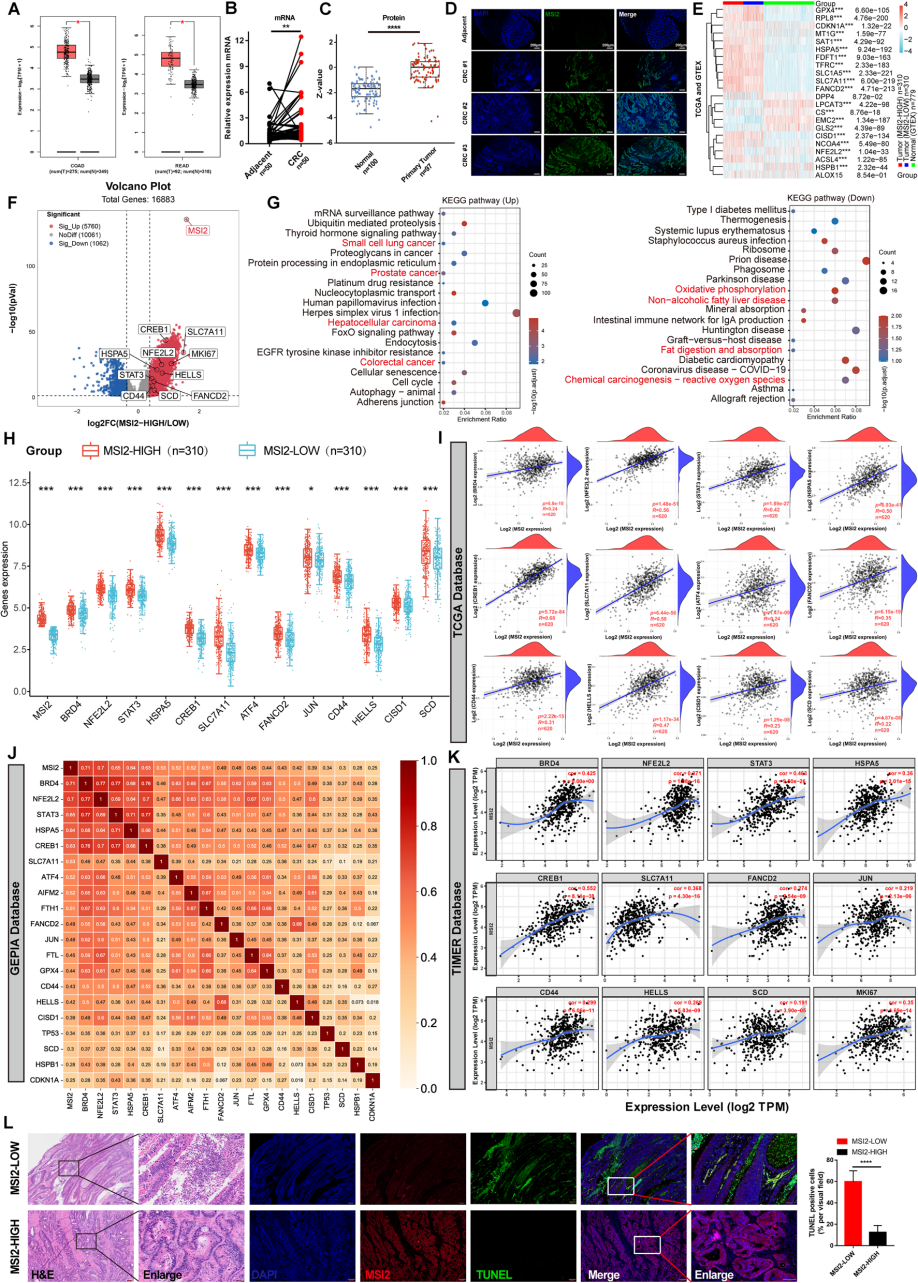
MSI2 expression is increased in CRC and positively correlated with the expression of ferroptosis-related inhibitory molecules. **A** The expression of MSI2 is upregulated in the CRC TCGA database from GEPIA (COAD, *N* = 349, T = 275; READ, *N* = 318, T = 92). **B** MSI2 mRNA expression was measured by qRT‒PCR in 50 paired clinical CRC specimens. **C** MSI2 protein expression was increased in colon primary tumors from CPTAC (*N* = 100, T = 97). **D** Representative IFC images of MSI2 expression in our clinical CRC specimens. Blue: DAPI, green: MSI2; Scale bars, 200 μm. **E** Heatmap of ferroptosis-related gene expression in MSI2-high (*n* = 310) and MSI2-low (*n* = 310) patients from TCGA CRC datasets and in normal tissues (*n* = 779) from GTEx. The different colors represent the expression trend of the indicated gene in different samples. **F** The volcano plot of the differentially expressed ferroptosis-related genes in MSI2-high (*n* = 310) and MSI2-low (*n* = 310) patients, “Adjusted *p* < 0.05 and Log2(Fold Change MSI2-high/MSI2-low) > 1.3 or < − 1.3” were defined as the threshold for DEGs. **G** The top up-regulated and down-regulated KEGG pathway enriched by the differentially expressed genes in MSI2-high (*n* = 310) and MSI2-low (*n* = 310) patients. **H** The mRNA expression distribution of ferroptosis-related inhibitory molecules in MSI2-high (*n* = 310) and MSI2-low (*n* = 310) patients. The abscissa represents differentially expressed ferroptosis-related inhibitory genes, and the ordinate represents the expression distribution of genes. **I** The positive correlations between MSI2 and ferroptosis-related inhibitory genes were analyzed by *Spearman* correlation analysis in the TCGA CRC datasets, *n* = 620. **J** Heatmap of positive correlations between MSI2 and ferroptosis-related inhibitory genes were analyzed by *Spearman* correlation analysis and normalized by GAPDH in the GEPIA COAD and READ database, *n* = 367. **K** The positive correlations between MSI2 and ferroptosis-related inhibitory genes were analyzed by *Spearman* correlation analysis in the TIMER COAD database, *n* = 457. The ferroptosis-related genes set (**H-K**) were obtained from the public available FerrDb databases (http://www.zhounan.org/ferrdb/current/). **L** Representative images of necrotic cell death and TUNEL staining in MSI2-high and MSI2-low clinical CRC adenocarcinoma tissues. Blue: DAPI, red: MSI2, green: TUNEL; H&E, Scale bars, 200 μm (left) and 50 μm (right); IFC, Scale bars, 200 μm and 100 μm. and statistical analysis of TUNEL-positive cell percentages from MSI2-high and MSI2-low adenocarcinoma tissues. These results are presented as the mean ± SD values; *p < 0.05, **p < 0.01, ***p < 0.001, ****p < 0.0001; **B** paired 2-tailed Student’s t test, **A**, **C**, **H** Wilcox test, **E** Kruskal‒Wallis test, **I-K** *Spearman* correlation analysis and **L** unpaired 2-tailed Student’s t test

Currently, data describing the effects of MSI2 on tumor initiation and progression have been extensively explored [19, 21]. However, no study has focused on the role of MSI2 in tumor iron death, particularly in CRC. To explore the ferroptosis effect of MSI2 on CRC, we first analyzed MSI2 expression through the TCGA CRC datasets via the GEPIA tool and found that the transcript level of MSI2 was higher in tumor tissue than in normal tissue (Fig. 1A). Consistently, compared with that in adjacent normal colon tissues, the mRNA expression of MSI2 was upregulated in our 50 pairs of clinical CRC specimens (Fig. 1B). Elevated protein levels of MSI2 were also found in CRC primary tumors compared to the normal tissues in the CPTAC datasets, which was further confirmed by immunofluorescence histological examination of our clinical CRC patient samples (Fig. 1C and D). Next, to clarify the association between MSI2 and ferroptosis in CRC, we collected 620 CRC patients from the TCGA database and 779 normal tissues from GTEx. And CRC patients were classified into high and low expression groups based on MSI2 transcript levels, and we observed that the expression of GPX4, SLC7A11, FANCD2, NFE2L2 and HSPA5 was upregulated in CRC patients (Fig. 1E). Among them, the transcriptional expression of ferroptosis inhibitor molecules was upregulated in MSI2-High patients, such as SLC7A11, NFE2L2, HSPA5, FANCD2, HELLS, etc. (Fig. 1F). And KEGG pathway enrichment also showed that the differentially expressed genes involved in the small cell lung cancer, prostate cancer, hepatocellular cancer and colorectal cancer pathways were significantly up-regulated, while genes involved in the oxidative phosphorylation, non-alcoholic fatty liver disease, fat digestion and absorption, chemical carcinogenesis-reactive oxygen species and neuro-degenerative disease pathways were significantly down-regulated (Fig. 1G). Notably, MSI2 expression was significantly associated with ferroptosis inhibitor molecules, which were upregulated in patients with high MSI2 expression; including BRD4, NFE2L2, STAT3, HSPA5, CREB1, SLC7A11, ATF4, FANCD2, JUN, CD44, HELLS, CISD1 and SCD (Fig. 1H). Furthermore, the positive correlations between MSI2 and ferroptosis inhibitor genes in TCGA CRC datasets were further identified, and the positively correlated genes included BRD4 (*R* = 0.24, *p* = 6.8e-10), NFE2L2 (*R* = 0.56, *p* = 1.48e-51), STAT3 (*R* = 0.42, *p* = 1.89e-27), HSPA5 (*R* = 0.50, *p* = 6.93e-41), CREB1 (*R* = 0.68, *p* = 5.72e-84), SLC7A11 (*R* = 0.58, *p* = 6.44e-56), ATF4 (*R* = 0.24, *p* = 1.47e-09), FANCD2 (*R* = 0.35, *p* = 6.15e-19), CD44 (*R* = 0.31, *p* = 2.22e-15), HELLS (*R* = 0.47, *p* = 1.17e-34), CISD1 (*R* = 0.23, *p* = 1.29e-08) and SCD (*R* = 0.22, *p* = 4.87e-08) (Fig. 1I). Similarly, the positive correlations between MSI2 and ferroptosis inhibitor genes were also confirmed in the GEPIA and TIMER CRC datasets (Fig. 1J and K). More importantly, our TUNEL staining and histological analysis revealed that clinical CRC samples with low MSI2 expression exhibited more tissue necrotic cell death and higher rates of TUNEL-positive staining than those with high MSI2 expression (Fig. 1L). Collectively, these findings suggest that MSI2 expression is positively correlated with the expression of ferroptosis inhibitor genes, which may control iron death in CRC.

### MSI2 Deficiency Represses the Proliferation, Migration and Invasion of CRC Cells In Vitro

**Fig. 2:**
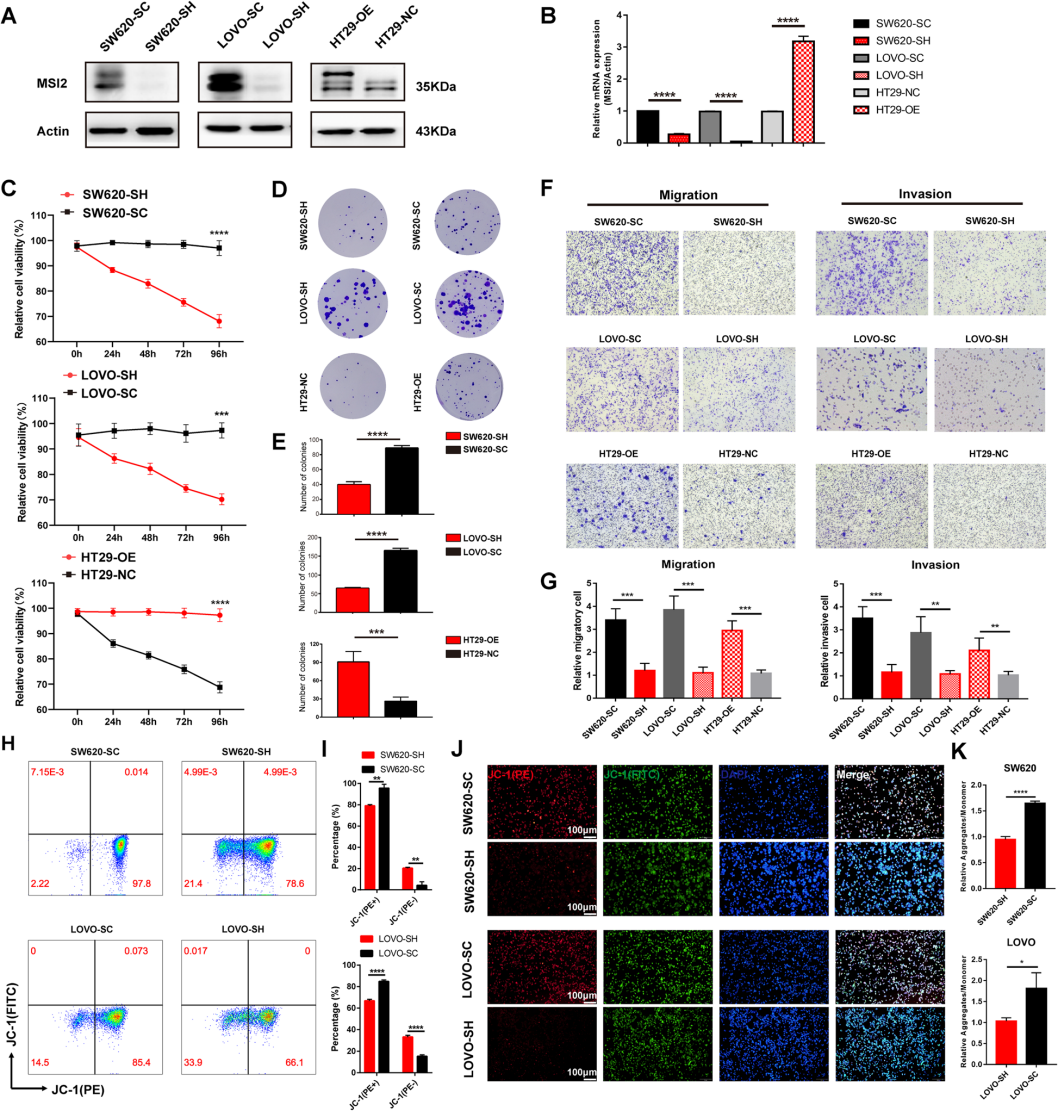
MSI2 deficiency inhibits the proliferation, migration and invasion of CRC cells in vitro. **A** Western blotting for MSI2 expression in SW620, LOVO, and HT29 stable cells. **B** MSI2 mRNA expression was determined by qRT‒PCR in SW620, LOVO, and HT29 stable cells. **C**, The cell viability and proliferation were monitored by CCK-8 at 0–96 h in SW620, LOVO, and HT29 stable cells. **D-E** Representative images and statistical analysis of the colony formation assay of stable cell lines showed that MSI2 deficiency significantly reduced colony formation. **F-G** Representative images and statistical analysis of Transwell migration and invasion of SW620, LOVO, and HT29 stable cell lines showed that MSI2 deficiency significantly inhibited migration and invasion. **H** FACS analysis of the mitochondrial membrane potential assay with JC-1 showed that MSI2 deficiency significantly increased JC-1 monomer (PE^−^ fluorescence) percentages. **I** Statistical FACS analysis percentages of JC-1 monomers (PE^−^ fluorescence) and JC-1 aggregates (PE^+^ fluorescence) in SW620 and LOVO stable cells. **J** Representative IFC images of the mitochondrial membrane potential assay with JC-1 in SW620 and LOVO stable cells. Blue: DAPI, red: JC-1 (PE), green: JC-1 (FITC); Scale bars, 200 μm. **K** Statistical IFC analysis of JC-1 aggregates and JC-1 monomers in SW620 and LOVO stable cells. These results are presented as the mean ± SD values; *p < 0.05, ***p < 0.001, ****p < 0.0001; **B**, **C**, **E**,** G**,** I**,** K** unpaired 2-tailed Student’s t test

To clarify how MSI2 modulates CRC functions, we first examined the expression of MSI2 mRNA and protein in CRC cell lines and found that the MSI2 protein was upregulated in SW620 and LOVO cells but downregulated in HT29 cells (Fig. S1A and B). Therefore, we constructed stable cell lines and verified the expression of MSI2 protein and mRNA by Western blotting and qRT‒PCR, respectively (Fig. 2A and B). Furthermore, by using DepMap analysis of the CCLE datasets, the gene effect scores of MSI2 were also assessed in colorectal cancer cell lines and revealed that almost all the CRC cell line scores were less than 0 (Fig. S1C), and these negative scores implied cell growth inhibition and/or cell death following MSI2 gene knockout in each cell line. To determine whether MSI2 affects CRC cell proliferation, we first performed CCK-8 assays to assess the viability of stable cells after MSI2 knockdown in SW620 and LOVO cells or overexpression in HT29 cells and found that MSI2 increased the viability of stable cells in vitro (Fig. 2C). The cell cloning ability was further detected by colony formation assays, and the results indicated that MSI2 deficiency significantly suppressed the proliferation and colony formation of stable cells in vitro (Fig. 2D and E). Moreover, in vitro cell migration and invasion assays were performed to explore whether MSI2 knockdown could inhibit the migration and invasion of CRC cells. And the migration and invasion of tumor cells were markedly suppressed after MSI2 knockdown in SW620 and LOVO stable cells but significantly promoted in HT29 stable cells upon MSI2 overexpression (Fig. 2F and G). Mitochondrial membrane potential (MMP) is the key indicator of mitochondrial function that controls cell viability and death. We further used the MMP Assay Kit with JC-1 to detect the monomer and aggregates of MMP by FACS to quantify MMP changes after MSI2 knockdown. The percentages of JC-1 aggregates (PE^+^) in stable SW620 and LOVO cells were clearly decreased after MSI2 knockdown, whereas JC-1 monomer (PE^−^) percentages were increased (Fig. 2H and I). Consistent with this finding, an immunofluorescence assay with JC-1 staining of stable SW620 and LOVO cells confirmed that the JC-1 aggregate (PE^+^) intensity was decreased after MSI2 knockdown; the shift from aggregates to monomers indicates the loss of MMP in MSI2-deficient CRC cells (Fig. 2J and K). Taken together, these data suggest that MSI2 deficiency decreases the viability and inhibits the proliferation, migration and invasion of CRC cells in vitro.

### MSI2 Deficiency Promotes Ferroptosis in CRC Cells in Vitro

**Fig. 3:**
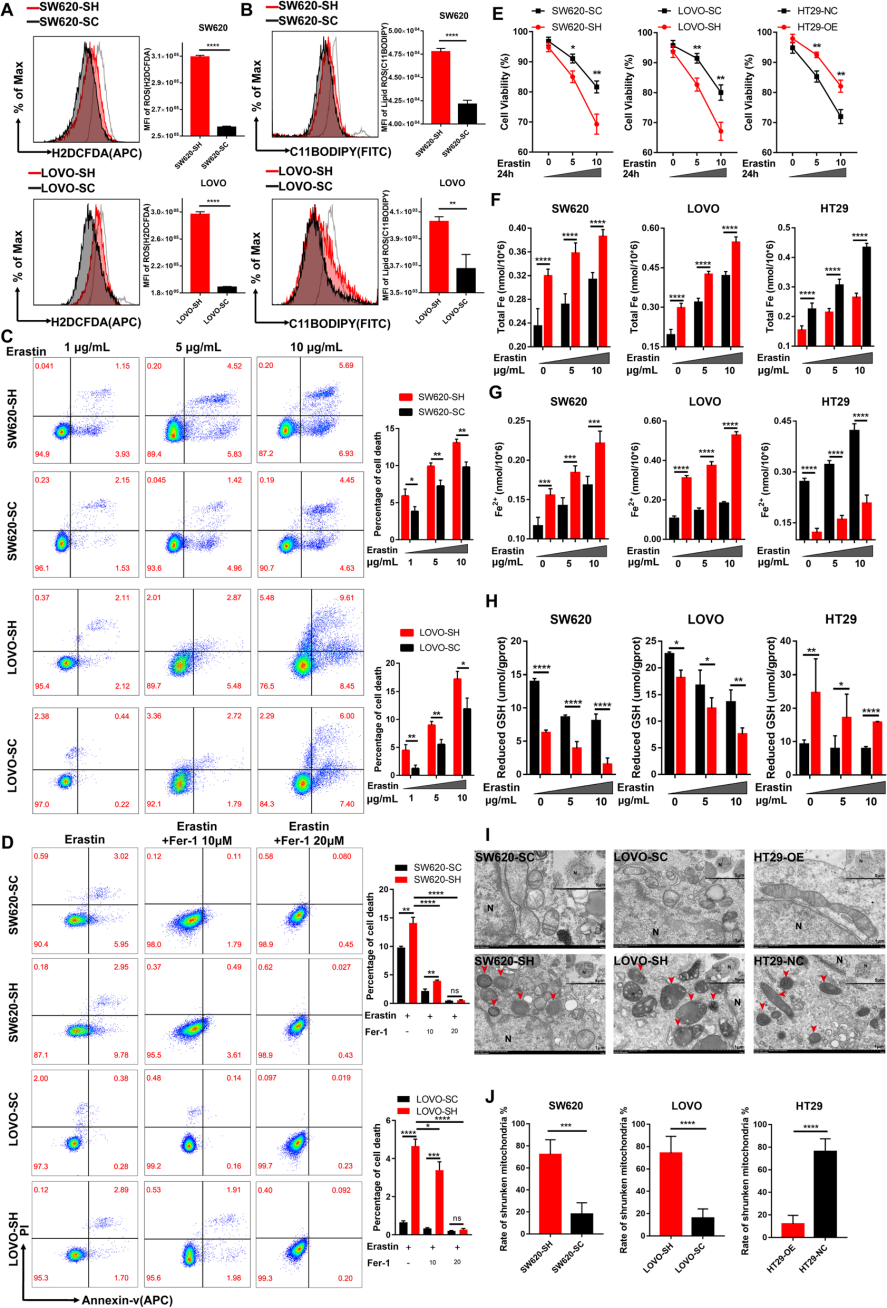
MSI2 deficiency promotes the ferroptosis of CRC cells in vitro. **A** FACS and statistical analysis of total ROS levels (H2DCFDA) in SW620 and LOVO stable cells, gray indicates the positive control. **B** FACS and statistical analysis of lipid-ROS levels (C11-BODIPY) in SW620 and LOVO stable cells, gray indicates the positive control. **C**, FACS and statistical analysis of cell death rate in SW620 and LOVO stable cells after treatment with erastin (1–10 µg/mL) for 12 h. **D**, FACS and statistical analysis of cell death rate in SW620 and LOVO stable cells after treatment with erastin (5 µg/mL) and Fer-1 (10 and 20 µM) for 8 h. **E** The cell viability was monitored by CCK-8 and treated with different concentrations of erastin for 24 h in stable SW620, LOVO and HT29 cells. **F** The levels of intracellular ferrous ions were determined by treatment with different concentrations of erastin for 8 h in stable SW620, LOVO and HT29 cells. **G** The levels of intracellular total iron were determined by treatment with different concentrations of erastin for 8 h in stable SW620, LOVO and HT29 cells. **H** The levels of intracellular reduced GSH were determined by treating SW620, LOVO and HT29 stable cells with different concentrations of erastin for 8 h. **I** Representative images of electron microscopy revealed obvious mitochondrial abnormalities in MSI2-deficient stable cells. Scale bars, 5 μm (up) and 1 μm (bottom). **J** Statistical analysis of the shrunken mitochondria rate in SW620, LOVO and HT29 stable cells. These results are presented as the mean ± SD values; *p < 0.05, **p < 0.01, ***p < 0.001, ****p < 0.0001; **A-H**,** J** unpaired 2-tailed Student’s t test

As we described above, MSI2 was positively correlated with ferroptosis inhibitor genes, promoting cell proliferation and suppressing MMP collapse. We wondered what effect MSI2 might have on iron death in CRC cells. We first examined the total reactive oxygen species (ROS) level of stable SW620 and LOVO cells by H2DCFDA staining and FACS analysis, we found that the total ROS levels were increased after MSI2 knockdown in SW620 and LOVO stable cells (Fig. 3A). As an iron-dependent form of cell death, ferroptosis is triggered by the accumulation of toxic lipid reactive oxygen species, particularly lipid hydroperoxides [51]. Next, the intracellular lipid ROS level was further detected using C11-BODIPY staining and FACS analysis. Notably, the lipid ROS levels were also significantly increased after MSI2 knockdown in SW620 and LOVO stable cells (Fig. 3B). Furthermore, to confirm the effect of MSI2 on cell death process, we used FACS to examine the cell mortality rates of stable SW620 and LOVO cells following incubation with different concentrations of erastin, an inducer of ferroptosis, for 12 h. We observed an increased percentage of cell death in the SW620 and LOVO MSI2 knockdown groups stable cell lines after erastin treatment (Fig. 3C), which indicates that MSI2 deficiency promotes erastin-induced iron death. However, it was found that there was no significant difference in SW620 and LOVO cell mortality between the SC and SH groups without any treatment (Fig. S2A), as well as the mRNA expression levels of apoptosis-related genes after MSI2 knockdown in SW620 and LOVO stable cells, including BCL2, BAX, CASP3 and CASP8 (Fig. S2B). And there was also inconsistency in the expression association analysis between MSI2 and pro-apoptotic and anti-apoptotic genes, such as BCL2 and CASP3 (Fig. S2C). These findings are consistent with a recent study that the loss of MSI2 does not affect the apoptosis of CRC cells [31]. More importantly, Ferrostatin 1 (Fer-1) is a potent inhibitor of ferroptosis, which can inhibit erastin-induced ferroptosis and reduce the accumulation of lipid peroxides. Therefore, FACS analysis of SW620 and LOVO stable cells showed that erastin and MSI2-induced cell iron death could be reversed by ferroptosis inhibitor Fer-1 (Fig. 3D), further indicating that MSI2 deficiency triggers CRC ferroptosis rather than cell apoptosis. Moreover, the cell viability was remarkably reduced in the SW620 and LOVO MSI2 knockdown groups after incubation with increasing dose of erastin. Conversely, the cell viability was significantly increased in HT29-OE group cells compared to NC group cells (Fig. 3E). In addition, we also determined the levels of intracellular ferrous iron and total iron in stable cells and found that the total iron and ferrous iron concentrations were both increased after incubation with different concentrations of erastin. Importantly, the intracellular ferrous iron and total iron levels were both significantly increased in SW620-SH and LOVO-SH group cells compared to SC group cells but significantly downregulated in HT29-OE group cells compared to NC group cells (Fig. 3F and G). Intracellular reduced glutathione (GSH) is an important indicator of the cellular redox state [52]. Consistent with the above finding, after treatment with different concentrations of erastin, we found that the intracellular reduced GSH levels were both significantly decreased in SW620-SH and LOVO-SH group cells compared to SC group cells but increased in HT29-OE group cells compared to NC group cells (Fig. 3H), which indicates that the cellular redox state in MSI2 knockdown cells markedly shifts from the reduced state to the oxidized state. More importantly, we used transmission electron microscopy to evaluate the mitochondrial morphology of stable cells. The SW620-SH, LOVO-SH and HT29-NC group cells had obvious mitochondrial abnormalities, and the rate of shrunken mitochondria was significantly increased after MSI2 knockdown in SW620 and LOVO stable cells, but decreased in HT29 cells upon MSI2 overexpression (Fig. 3I and J). Together, these data suggest that MSI2 deficiency triggers ferroptosis in CRC cells in vitro.

### MSI2 Deficiency Triggers CRC Ferroptosis by Downregulating the MAPK Signaling Cascade to Inhibit HSPB1 Phosphorylation

**Fig. 4:**
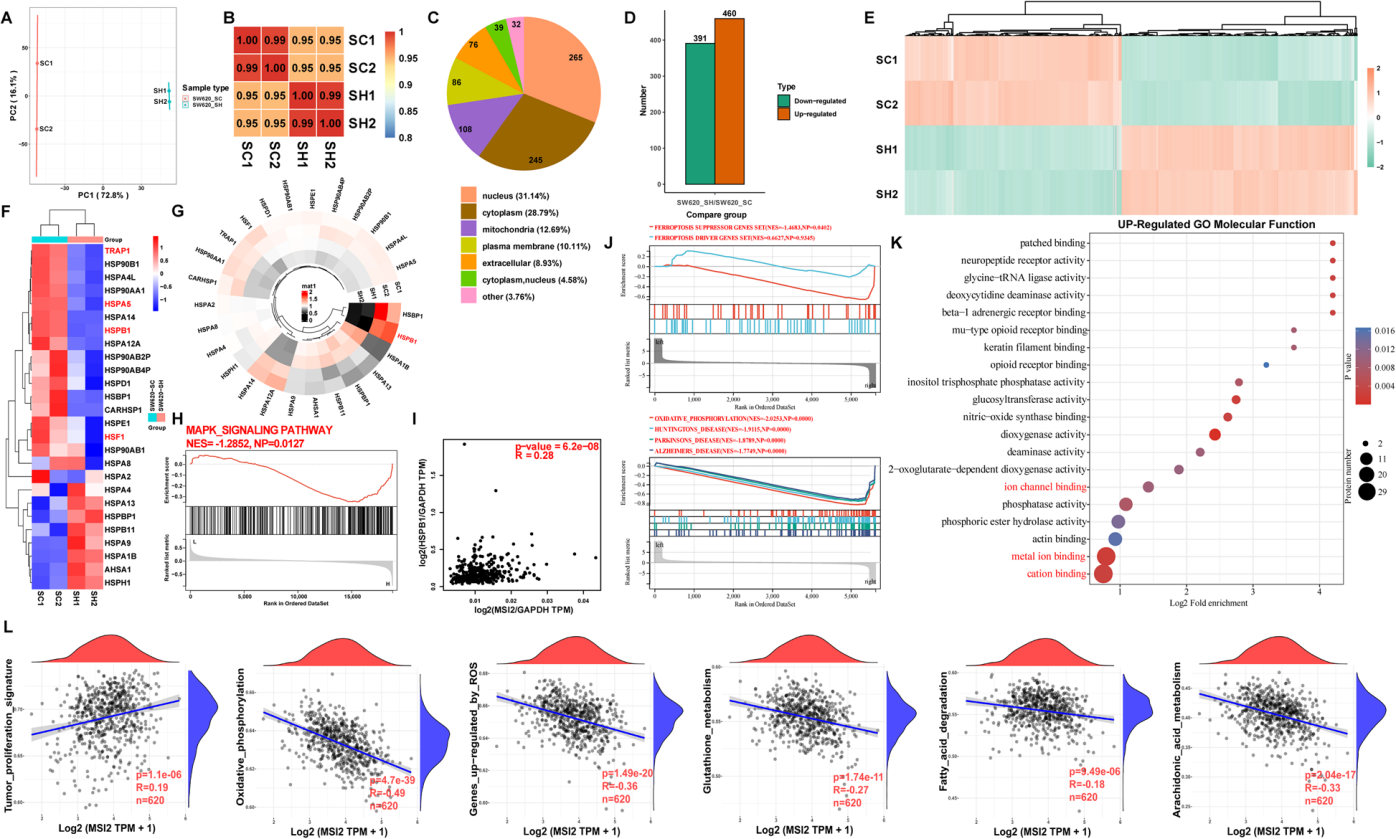
MSI2 dysregulates HSP family gene expression and multiple signal transduction processes. **A** Principal component analysis (PCA) plot of stable SW620-SC and SW620-SH cells in the proteomics analysis. **B** *Pearson* correlation analysis between stable SW620-SC and SW620-SH cells in the proteomics analysis. **C** The distribution, localization and proportion of significantly genes corresponding to differentially expressed proteins in SW620 cell proteomics. **D** In total, 460 upregulated protein genes and 391 downregulated protein genes were identified. **E** Heatmap of all the genes corresponding to differentially expressed proteins identified by proteomics. **F** Heatmap of differential HSP family protein genes identified in proteomics. **G** Circular heatmap demonstrating the expression of the most differentially expressed HSP family protein gene in proteomics. **H** The downregulated Gene set enrichment analysis (GSEA) of KEGG pathway was enriched in the MAPK signaling pathway. **I**, The positive correlation between MSI2 expression and HSPB1 expression was analyzed by *Spearman* correlation analysis and normalized by GAPDH in the GEPIA COAD and READ database (*p* = 6.2e-08, *R* = 0.28), *n* = 367. **J** GSEA analysis of differential proteins revealed the most significantly downregulated KEGG pathways, such as oxidative phosphorylation, Huntington’s disease, Parkinson’s disease, Alzheimer’s disease and ferroptosis suppressor genes set, while the ferroptosis driver genes set was upregulated, and ferroptosis-related genes set were obtained from FerrDb databases. **K** The up-regulated GO molecular functions (MFs) were enriched in metal ion binding and ion channel binding. **L** Associations between MSI2 expression and aberrant signaling pathway score from TCGA CRC datasets (*n* = 620), such as the positive correlation with tumor proliferation signature (*R* = 0.19), the negative correlations with genes upregulated by ROS (*R*=-0.36) and glutathione metabolism (*R*=-0.27)

To determine how MSI2 regulates CRC ferroptosis, we performed 4D label-free proteomics analysis in SW620 stable cells to identify the genes corresponding to differentially expressed proteins (Fig. 4A and B). After MSI2 knockdown, the genes corresponding to differentially expressed protein included 391 downregulated genes and 460 upregulated genes, of which 265 (31.14%) were located in the nucleus, 245 (28.79%) were located in the cytoplasm, and 108 (12.69%) were located in the mitochondria (Fig. 4C-E). HSPs, which function as molecular chaperones, have been found to be involved in a variety of cellular stress, redox homeostasis and iron cell death processes, especially aberrant transduction involving the MAPK signaling pathway [33]. We then analyzed the differentially expressed HSP family genes and found that the genes encoding proteins such as HSPA5, HSPB1, TRAP1, HSF1 and HSP90 were downregulated after MSI2 knockdown, while the genes encoding proteins such as HSPH1 and HSP1AB were upregulated (Fig. 4F). Previous studies have demonstrated the pivotal inhibitory effects of HSPA5, HSPB1, HSF1 and HSPH1 in cancer ferroptosis [13, 34, 53]. Notably, HSPB1 was markedly downregulated in SW620-SH group cells following MSI2 knockdown (Fig. 4G). In addition, HSPB1, is one of the important downstream targets for MAPK signal transduction to control cell death and proliferation, GSEA enrichment analysis further revealed that the MAPK signaling pathway (NES=-1.2852) was downregulated after MSI2 knockdown (Fig. 4H). Consistent with this finding, the expression of HSPB1 was significantly positively correlated with that of MSI2 in the GEPIA CRC datasets (*p* = 6.2e-08, *R* = 0.28) (Figs. 1I and 4I). Consistent with the results from TCGA in Fig. 1G, GSEA enrichment analysis of SW620 proteomics also suggested that MSI2 was involved in the regulation of other important signaling pathways, such as oxidative phosphorylation (NES=-2.0253), Huntington’s disease (NES=-1.9115), Parkinson’s disease (NES=-1.8789) and Alzheimer’s disease (NES=-1.7749) (Fig. 4J), and recent studies have also discovered that these neuro-degenerative diseases exhibited the key features of ferroptosis: lipid peroxidation and iron accumulation [54]. More importantly, GSEA enrichment analysis of ferroptosis-related genes set suggested that the suppressor genes set (NES=-1.4683) was significantly downregulated after MSI2 knockdown, while the driver genes set (NES = 0.6627) was not significantly upregulated (Fig. 4J). Also, enriched up-regulated GO molecular functions (MFs) included cation binding, metal ion binding and ion channel binding (Fig. 4K). Moreover, the associations between MSI2 expression and multiple signaling pathways score from TCGA CRC database were further analyzed, and it was found that MSI2 positively correlated with tumor proliferation signature (*p* = 1.1e-06, *R* = 0.19), but negatively correlated with oxidative phosphorylation (*p* = 4.7e-39, *R*=-0.49), genes upregulated by ROS (*p* = 1.49e-20, *R*=-0.36), glutathione metabolism (*p* = 1.74e-1, *R*=-0.27), Fatty acid degradation (*p* = 9.49e-06, *R*=-0.18) and arachidonic acid metabolism (*p* = 2.04e-17, *R*=-0.33) (Fig. 4L). Furthermore, the expression of pro-apoptotic genes CASP3 and BAX protein was down-regulated in the SW620-SH group, while the expression of CASP8, BCL2L1 and BCL2L13 protein was up-regulated. The apoptosis signaling pathway in GSEA enrichment analysis also did not show significant differences, indicating that the apoptotic effect of MSI2 on CRC is not particularly pronounced (Fig. S2D and E).

**Fig. 5:**
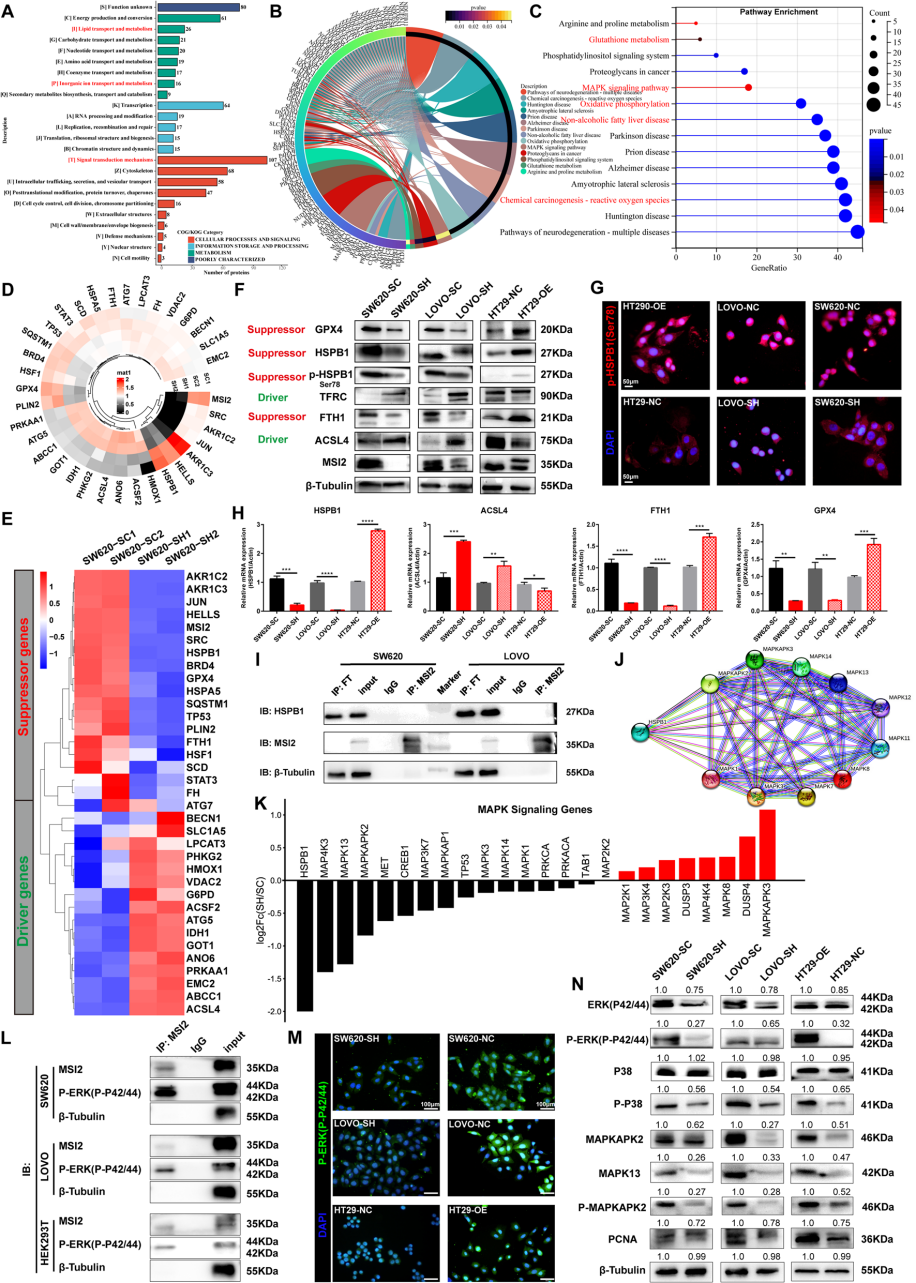
MSI2 deficiency triggers CRC ferroptosis by downregulating the MAPK signaling cascade to inhibit HSPB1 phosphorylation. **A** The functions of genes corresponding to differentially expressed proteins were annotated based on KOG/COG, and the main enriched metabolic pathways were lipid transport and metabolism and inorganic ion transport and metabolism. **B-C** Genes corresponding to the differentially expressed proteins were functionally annotated based on the KEGG database and were mainly enriched in nonalcoholic fatty liver disease, oxidative phosphorylation, chemical carcinogenesis-reactive oxygen species and MAPK signaling pathway. **D** Circular heatmap demonstrating the most significant differential expression of ferroptosis-related genes in stable SW620 cell proteomics analysis. **E** Heatmap showing the differential expression of ferroptosis-related driver or suppressor genes in stable SW620 cell proteomics analysis. **F** Verification of differential gene expression in SW620, LOVO, and HT29 stable cells by using Western blotting for MSI2, HSPB1, p-HSPB1(Ser78), ACSL4, FTH1, TFRC and GPX4. **G** Representative IFC images of p-HSPB1(Ser78) expression in SW620, LOVO and HT29 stable cells. Blue: DAPI, red: p-HSPB1(Ser78); Scale bars, 50 μm. **H** HSPB1, ACSL4, FTH1 and GPX4 genes mRNA expression were determined by qRT‒PCR in SW620, LOVO, and HT29 stable cells. **I** Co-immunoprecipitation assays showed that there was no direct interaction between MSI2 and HSPB1 in SW620 and LOVO cells. FT means flow through wash fraction, input and anti-IgG as controls. **J** The protein–protein interaction (PPI) networks between HSPB1 and MAPK family genes were constructed by STRING. **K** Differential MAPK signaling pathway genes expression in stable SW620 cell proteomics analysis. **L** Co-immunoprecipitation assays showed that MSI2 could interact with p-ERK(p-P42/44) in SW620, LOVO and HEK293T cells. The input and anti-IgG as controls. **M** The protein levels of p-ERK(p-P42/44) were determined by IFC in SW620, LOVO and HT29 stable cells. Blue: DAPI, green: p-ERK(p-P42/44); Scale bars, 50 μm. **N** Western blotting for MAPK signaling cascade gene expression, such as ERK(P42/44), p-ERK(p-P42/44), P38, p-P38, MAPKAPK2, MAPK13, p-MAPKAPK2 and PCNA, in SW620, LOVO, and HT29 stable cells. These results are presented as the mean ± SD values; *p < 0.05, **p < 0.01, ***p < 0.001, ****p < 0.0001; **H** unpaired 2-tailed Student’s t test

Next, to fully elucidate the underlying mechanism by which MSI2 regulates ferroptosis, COG/KOG and KEGG pathway enrichment analyses were performed to assess the dysregulation of signal transduction. Among the differentially expressed genes, 26 genes were involved in lipid transport and metabolism processes, and 16 genes were involved in inorganic ion transport and metabolism processes (Fig. 5A). KEGG pathway analysis also showed the other abnormal enrichment of non-alcoholic fatty liver disease, oxidative phosphorylation, glutathione metabolism and chemical carcinogenesis-reactive oxygen species pathways (Fig. 5B and C). Through FerrDb database and SW620 cells proteomics, we further analyzed the expression of ferroptosis driver and suppressor genes and found that the driver genes, such as HMOX1, ACSL4, ACSF2, ATG5, EMC2 and GOT1, were upregulated after MSI2 knockdown. However, the suppressor genes, such as HSPB1, HELLS, BRD4, AKR1C2, JUN, SRC, GPX4 and FTH1, were downregulated after MSI2 knockdown (Fig. 5D and E). More importantly, the identified differentially expressed genes were further validated by Western blotting, and the expression of HSPB1, p-HSPB1(Ser78), FTH1 and GPX4 was decreased after MSI2 knockdown, while the expression of TFRC and ACSL4 was increased (Fig. 5F). The downregulation of phosphorylated HSPB1 (Ser78) was further confirmed by immunofluorescence in HT29-NC, SW620-SH and LOVO-SH group cells (Fig. 5G). Consistently, the mRNA levels of HSPB1, FTH1 and GPX4 were decreased after MSI2 knockdown, while ACSL4 levels were increased (Fig. 5H). We wondered whether MSI2 could directly interact with HSPB1 protein, and the co-immunoprecipitation results showed that there was no direct interaction between MSI2 and HSPB1 at the protein level (Fig. 5I). Moreover, the PPI networks of HSPB1 were collected from STRING, and HSPB1 was found to be closely linked with MAPK family genes, such as MAPKAPK2, MAPK1, MAPKAPK3 and MAPK13 (Fig. 5J). Previous studies have revealed the important functions of MAPKAPK2-HSPB1 axis phosphorylation in tumor progression and oxidative stress injury [46–49]. Similarly, as shown in Figs. 4H and 5B and C, abnormal signal transduction also occurred in the MAPK signaling pathway. Therefore, we further evaluated the differentially expressed MAPK signal transduction genes by proteomics analysis of SW620 stable cells, and the protein expression of MAPKAPK2 and MAPK13 was significantly decreased after MSI2 knockdown (Fig. 5K). Additionally, evidence for crosstalk between MSI2 and p-ERK(P42/44) has been observed in pancreatic cancer and leukemic cells [55, 56]. Intriguingly, we next determined the direct interaction between MSI2 and p-ERK(P42/44) in CRC cells and HEK293T cells by co-immunoprecipitation (Fig. 5L). And the downregulation of p-ERK(P42/44) level was further validated in MSI2 knockdown stable CRC cells by IFC (Fig. 5M). Finally, we examined and confirmed by Western blotting that the protein expression of p-ERK(P42/44), p-P38, MAPKAPK2, p-MAPKAPK2, MAPK13 and PCNA was decreased in stable cells after MSI2 knockdown (Fig. 5N). In conclusion, these findings suggest that MSI2 deficiency triggers CRC ferroptosis by downregulating the MAPK signaling cascade to inhibit HSPB1 phosphorylation.

#### MSI2 Deficiency Restrains CRC Malignancy by Inhibiting MAPK/HSPB1 axis Phosphorylation In Vivo

**Fig. 6:**
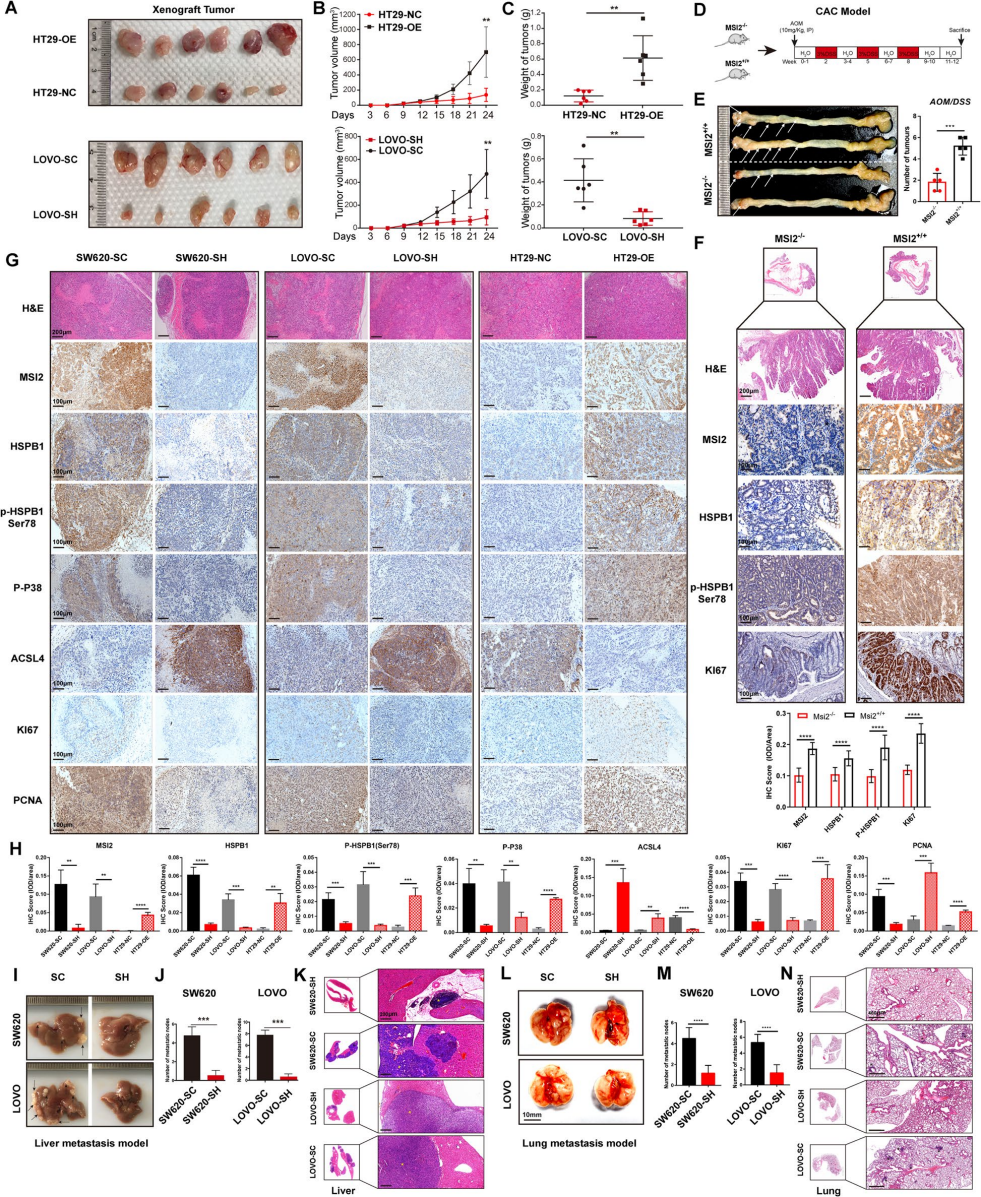
MSI2 deficiency suppresses CRC malignant features, including tumor proliferation and metastasis, by inhibiting MAPK/HSPB1 axis phosphorylation in vivo. **A** In vivo models of xenograft tumors from HT29 and LOVO stable cells were established in M-NSG mice, *n* = 6. **B** Quantitative analysis of xenografted tumor volume change from HT29 and LOVO stable cells, *n* = 6. **C**, Statistical analysis of xenografted tumor weight from HT29 and LOVO stable cells, *n* = 6. **D** Procedure for constructing colitis-associated colon cancer in vivo models of *MSI2*^*+/+*^ and *MSI2*^*−/−*^ transgenic mice. **E** Representative image and statistical analysis of colitis-associated colon cancer tumor number in *MSI2*^*+/+*^ and *MSI2*^*−/−*^ transgenic mice, *n* = 5. **F** Representative H&E, IHC images and statistical analysis of the IHC score (IOD per area) of MSI2, HSPB1, p-HSPB1(Ser78) and Ki67 in *MSI2*^*+/+*^ and *MSI2*^*−/−*^ transgenic CAC mice model. Scale bars, H&E, 200 μm; IHC, 100 μm. **G** Representative H&E and IHC images of xenograft tumors from SW620, LOVO and HT29 stable cells, including MSI2, HSPB1, p-HSPB1(Ser78), p-P38, ACSL4, Ki67 and PCNA. Scale bars, H&E, 200 μm; IHC, 100 μm. **H** Statistical analysis of the IHC score (IOD per area) of MSI2, HSPB1, p-HSPB1(Ser78), p-P38, ACSL4, Ki67 and PCNA from SW620, LOVO and HT29 stable cells xenograft tumors. **I-J** Representative images and statistical analysis of liver metastasis nodules from SW620 and LOVO stable cells in M-NSG mice, *n* = 5. **K **Representative images of H&E staining of liver metastasis from SW620 and LOVO stable cells. The yellow star indicates the tumor area. Scale bars, 200 μm. **L-M** Representative images and statistical analysis of lung metastasis nodules from SW620 and LOVO stable cells in M-NSG mice, *n* = 5. **N** Representative images of H&E staining of lung metastasis from SW620 and LOVO stable cells. The dark star indicates the tumor area. Scale bars, 400 μm. These results are presented as the mean ± SD values; *p < 0.05, **p < 0.01, ***p < 0.001, ****p < 0.0001; **B**, **C**,** E**,** F**,** H**,** J**,** M** unpaired 2-tailed Student’s t test

To evaluate the effect of MSI2 on the biological behavior of CRC in vivo, we first established a CRC cell line xenograft model in M-NSG mice. We observed that the tumors formed by LOVO cells with stable MSI2 knockdown were consistently smaller and lighter than those formed by control cells, while tumors formed by HT29 cells with stable MSI2 overexpression showed the opposite trends, indicating that MSI2 knockdown can effectively inhibit tumor growth in vivo (Fig. 6A-C). Next, we used MSI2 knockout (*MSI2*^*-/-*^) transgenic and wild-type (*MSI2*^*+/+*^) mice to construct a colitis-associated colon cancer model by administering azoxymethane (AOM) intraperitoneally and feeding with 3% DSS for three cycles (Fig. 6D). Consistent with the above findings, deletion of MSI2 markedly reduced the occurrence and number of tumors in the mouse colon (Fig. 6E). Analysis of IHC staining results for MSI2, HSPB1, p-HSPB1(Ser78) and Ki67 showed that MSI2 knockout could reduce the expression of HSPB1, p-HSPB1(Ser78) and the tumor proliferation capacity in transgenic CAC mice (Fig. 6F). Moreover, we performed histological staining analysis of xenografted mouse tumors, including H&E staining and IHC of SW620, LOVO and HT29 stable cells. Similarly, the IHC results showed that MSI2 knockdown significantly decreased the expression of HSPB1, p-HSPB1(Ser78), p-P38, Ki67 and PCNA and increased the expression of ACSL4 in SW620 and LOVO stable cells. Conversely, overexpression of MSI2 markedly increased the expression of HSPB1, p-HSPB1(Ser78), p-P38, Ki67 and PCNA and decreased the expression of ACSL4 in HT29 stable cells (Fig. 6G and H). In addition, we investigated the role of MSI2 in CRC metastasis in vivo. The establishment of CRC liver metastasis models was performed by injecting SW620 and LOVO stable cells into the M-NSG mouse spleen, and MSI2 knockdown significantly inhibited the liver metastasis of stable SW620 and LOVO cells, which was further confirmed by histological analysis (Fig. 6I-K). Similarly, the CRC lung metastasis model was constructed by injecting SW620 and LOVO stable cells into the tail veins of M-NSG mice, and the results showed that MSI2 knockdown also inhibited lung metastasis of stable SW620 and LOVO cells (Fig. 6L-N). All these data reveal that MSI2 deficiency suppresses the growth and metastasis of CRC in vivo by inhibiting MAPK/HSPB1 axis phosphorylation.

### HSPB1 Rescues the Phenotypes of MSI2 Deficiency on CRC Ferroptosis In Vitro and In Vivo

**Fig. 7:**
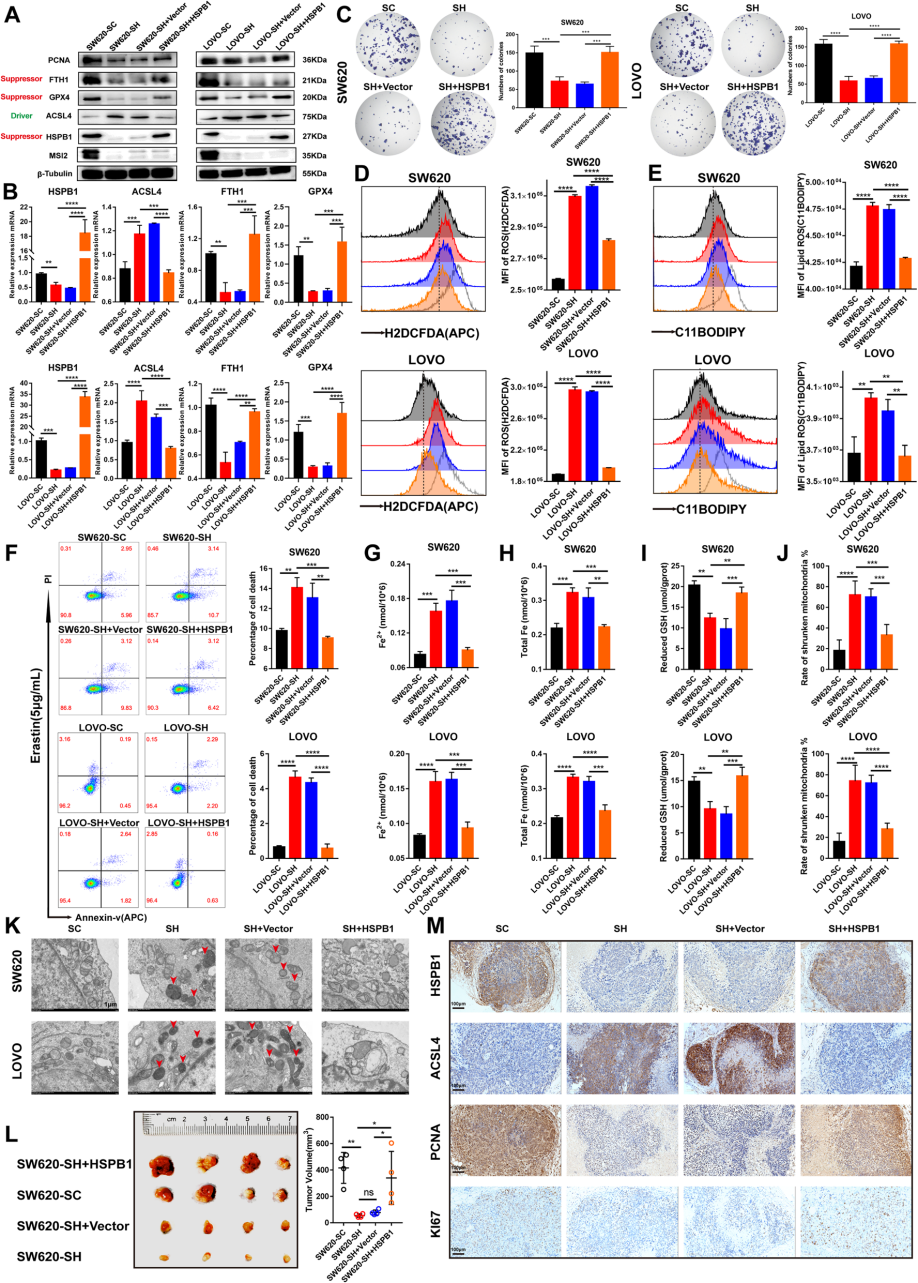
HSPB1 rescues the effect of MSI2 deficiency on CRC ferroptosis in vitro and in vivo. **A** Western blotting for MSI2, HSPB1, ACSL4, FTH1, PCNA and GPX4 expression in SW620 and LOVO stable cells rescued with vector or HSPB1 plasmids. **B** HSPB1, ACSL4, FTH1 and GPX4 mRNA expression was determined by qRT‒PCR in SW620 and LOVO stable cells rescued with vector or HSPB1 plasmids. **C** Representative images and statistical analysis of colony formation assays of stable SW620 and LOVO cells rescued with vector or HSPB1 plasmids. **D-E** FACS and statistical analysis of total ROS levels (H2DCFDA) (**D**) and lipid ROS levels (C11-BODIPY) (**E**) in SW620 and LOVO stable cells rescued with vector or HSPB1 plasmids, gray indicates the positive control. **F**, FACS and statistical analysis of cell death rate in SW620 and LOVO stable cells rescued with vector or HSPB1 plasmids and treated with erastin (5 µg/mL) for 8 h. **G-I** The levels of intracellular ferrous ions (**G**), total iron (**H**) and reduced GSH (**I**) were determined by treatment with erastin (10 µg/mL) for 8 h in SW620 and LOVO stable cells rescued with vector or HSPB1 plasmids. **J-K** Representative electron microscopy images and statistical analysis of mitochondrial abnormalities in stable SW620 and LOVO cells transfected with vector or HSPB1 rescue plasmids. Scale bars, 1 μm. **L** In vivo model of xenograft tumors from SW620 stable cells transfected with vector or HSPB1 rescue plasmids and statistical analysis of tumor volume in M-NSG mice, *n* = 4. **M** Representative IHC images of xenograft tumors from SW620 stable cells transfected with vector or HSPB1 rescue plasmids; these images show HSPB1, ACSL4, Ki67 and PCNA expression. Scale bars, 100 μm. These results are presented as the mean ± SD values; *p < 0.05, **p < 0.01, ***p < 0.001, ****p < 0.0001; **B-J**, **L** One-way ANOVA

To further address the role of the MSI2-HSPB1 axis in CRC ferroptosis, we investigated and determined whether the phenotypes of MSI2 deficiency could be reversed by rescuing HSPB1 expression during CRC ferroptosis. We first transfected the control vector and HSPB1 plasmid into SW620 and LOVO MSI2 knockdown cells to restore HSPB1 expression. The Western blotting results revealed that the protein expression of HSPB1, FTH1, PCNA and GPX4 was increased following rescue with the HSPB1 plasmid, but the expression of ACLS4 was decreased (Fig. 7A). Consistently, the mRNA expression levels of HSPB1, FTH1 and GPX4 were both upregulated after HSPB1 rescue, while ACLS4 expression was downregulated (Fig. 7B). Next, colony formation assays were performed to assess the capacity of CRC cell growth, and the results showed that HSPB1 rescue could improve the proliferation and colony formation ability of SW620 and LOVO MSI2 knockdown cells (Fig. 7C). Furthermore, we also examined intracellular total ROS and lipid-ROS levels after HSPB1 restoration in SW620 and LOVO MSI2 knockdown cells and found that the restoration of HSPB1 in MSI2 knockdown cells markedly reduced the levels of intracellular total ROS and lipid-ROS compared with those in the vector and untreated group (Fig. 7D and E). Similarly, the restoration of HSPB1 expression in SW620 and LOVO MSI2 knockdown cells also significantly reduced the cell death rate induced by erastin (Fig. 7F). More importantly, compared with those in the vector and untreated groups, the levels of intracellular ferrous iron and total iron were both decreased in the rescue group cells after HSPB1 transfection (Fig. 7G and H). However, in contrast, the intracellular reduced GSH levels were significantly increased in the rescue SW620-SH and LOVO-SH group cells compared to the vector group cells (Fig. 7I). In addition, by transmission electron microscopy, we observed that the mitochondrial morphology of the rescue group cells gradually recovered, and the proportion of shrunken mitochondria was significantly reduced after HSPB1 restoration in both SW620 and LOVO MSI2 knockdown stable cells (Fig. 7J and K). Moreover, we investigated whether restoring HSPB1 expression could reverse the tumor growth trend in vivo. Therefore, to validate our hypothesis, a xenograft tumor model in M-NSG mice was further constructed by injecting SW620 stable cells transfected with HSPB1, and we observed that restoration of HSPB1 expression significantly promoted tumor growth in vivo, including tumor volume and weight (Fig. 7L and S3A). Consistent with the above findings, in the IHC staining analysis of xenograft tumors, the restoration of HSPB1 expression in SW620 MSI2 knockdown cells markedly reduced the expression of ACSL4, but the expression of PCNA and Ki67 was increased (Fig. 7 M and S3B), indicating that HSPB1 could inhibit ferroptosis and promote CRC proliferation. Taken together, these results indicate that HSPB1 can rescue the effects of MSI2 deficiency on CRC ferroptosis in vitro and in vivo.

### High HSPB1 Expression Indicates a Poor Prognosis and HSPB1 is Positively Correlated with Ferroptosis Inhibitor Genes in CRC

**Fig. 8:**
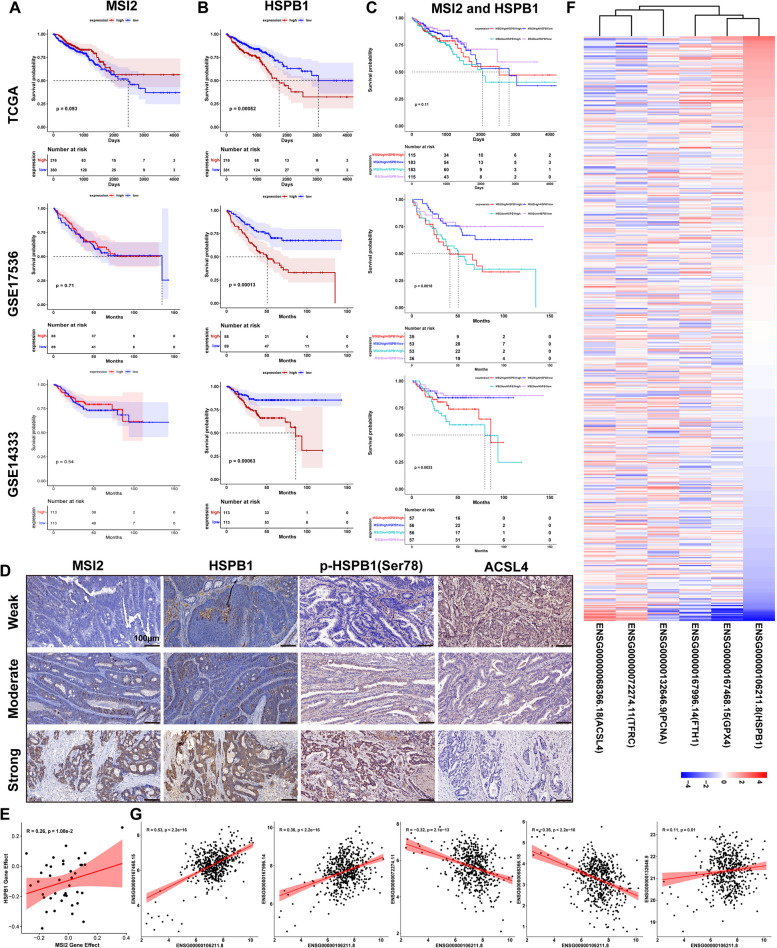
High HSPB1 expression indicates a poor prognosis in CRC patients. **A-B** Survival analysis of the HPA TCGA database (*n* = 596), GSE17536 (*n* = 177) and GSE14333 (*n* = 226) CRC datasets revealed that high or low MSI2 expression was not significantly associated with patient outcomes, but patients with high HSPB1 expression had poorer prognosis than those with low HSPB1 expression. **C** High MSI2 and HSPB1 expression indicates a poorer prognosis than low expression in the HPA TCGA database (*n* = 596), GSE17536 (*n* = 177) and GSE14333 (*n* = 226) CRC datasets. **D** Representative IHC images of MSI2, HSPB1, p-HSPB1(Ser78) and ACSL4 staining intensity in our clinical CRC specimens. Scale bars, 100 μm. **E** The positive gene effect *Pearson* correlation between MSI2 and HSPB1 from DEMETER2 CRC cell lines (*R* = 0.26). **F** Heatmap of HSPB1, GPX4, FTH1, PCNA, TFRC and ACSL4 expression from the GDC TCGA COAD database (*n* = 510). **G** *Pearson* correlation analysis from the GDC TCGA COAD database (*n* = 510) revealed that HSPB1 was positively correlated with the ferroptosis suppressor genes GPX4 (*R* = 0.53), FTH1 (*R* = 0.36) and PCNA (*R* = 0.11) but negatively correlated with the ferroptosis driver genes TFRC (*R*=-0.32) and ACSL4 (*R*=-0.36)

Finally, further survival analysis was performed to determine the prognostic impact of MSI2 and HSPB1 expression in CRC patients. The survival analysis showed that there was no significant difference in prognosis between CRC patients with high and low MSI2 expression in the CRC TCGA database (*p* = 0.093), GSE17536 (*p* = 0.71) and GSE14333 (*p* = 0.54) datasets (Fig. 8A). However, the patients with high HSPB1 expression had worse prognoses in the CRC TCGA database (*p* = 0.00052), GSE17536 (*p* = 0.00013) and GSE14333 (*p* = 0.00063) datasets (Fig. 8B), and the patients with lower HSPB1 expression had better prognoses. Moreover, survival analysis was performed by combining MSI2 and HSPB1 expression in CRC patients, and our results demonstrated that patients with high MSI2 and/or HSPB1 expression also had worse prognoses in the CRC TCGA (*p* = 0.11), GSE17536 (*p* = 0.0018) and GSE14333 (*p* = 0.0033) datasets (Fig. 8C). Furthermore, we also analyzed the expression intensity of MSI2, HSPB1, p-HSPB1(Ser78) and ACSL4 in our clinical CRC samples by IHC, and found that MSI2 expression was positively correlated with HSPB1 and p-HSPB1(Ser78) expression, but negatively correlated with ACSL4 expression (Fig. 8D), which was confirmed by the positive association between MSI2 and HSPB1 gene effect scores in CRC cell lines from DEMETER2 (*R* = 0.26) (Fig. 8E). In addition, previous studies have demonstrated that HSPB1 inhibits the ferroptosis process in various cancer cells [34–38], but its role in CRC has not been reported. Therefore, we further evaluated the associations between HSPB1 expression and other ferroptosis-related genes from the TCGA CRC database (Fig. 8F). We observed that HSPB1 expression was positively correlated with the expression of ferroptosis suppressor genes, such as GPX4 (*R* = 0.53), FTH1 (*R* = 0.36) and PCNA (*R* = 0.11) but negatively correlated with the expression of ferroptosis driver genes, such as ACSL4 (*R*=-0.36) and TFRC (*R*=-0.32) (Fig. 8G), suggesting that HSPB1 overexpression suppresses CRC ferroptosis. Collectively, these results indicate that patients with high MSI2 and HSPB1 expression have a poorer prognosis than those with low expression and that high expression of HSPB1 inhibits CRC ferroptosis.

## Discussion

Although an increasing number of cancer treatment options, such as traditional therapy surgery, neoadjuvant chemotherapy, radiotherapy, or targeted therapy, immunotherapy and combination therapies have improved patient survival, metastasis and recurrence are the major causes of the high mortality rate in CRC patients [57, 58]. The induction of ferroptosis holds great promise for cancer treatment [11–13]. Thus, identifying and targeting the underlying ferroptosis-related molecules represents an attractive therapeutic approach for CRC.

**Fig. 9:**
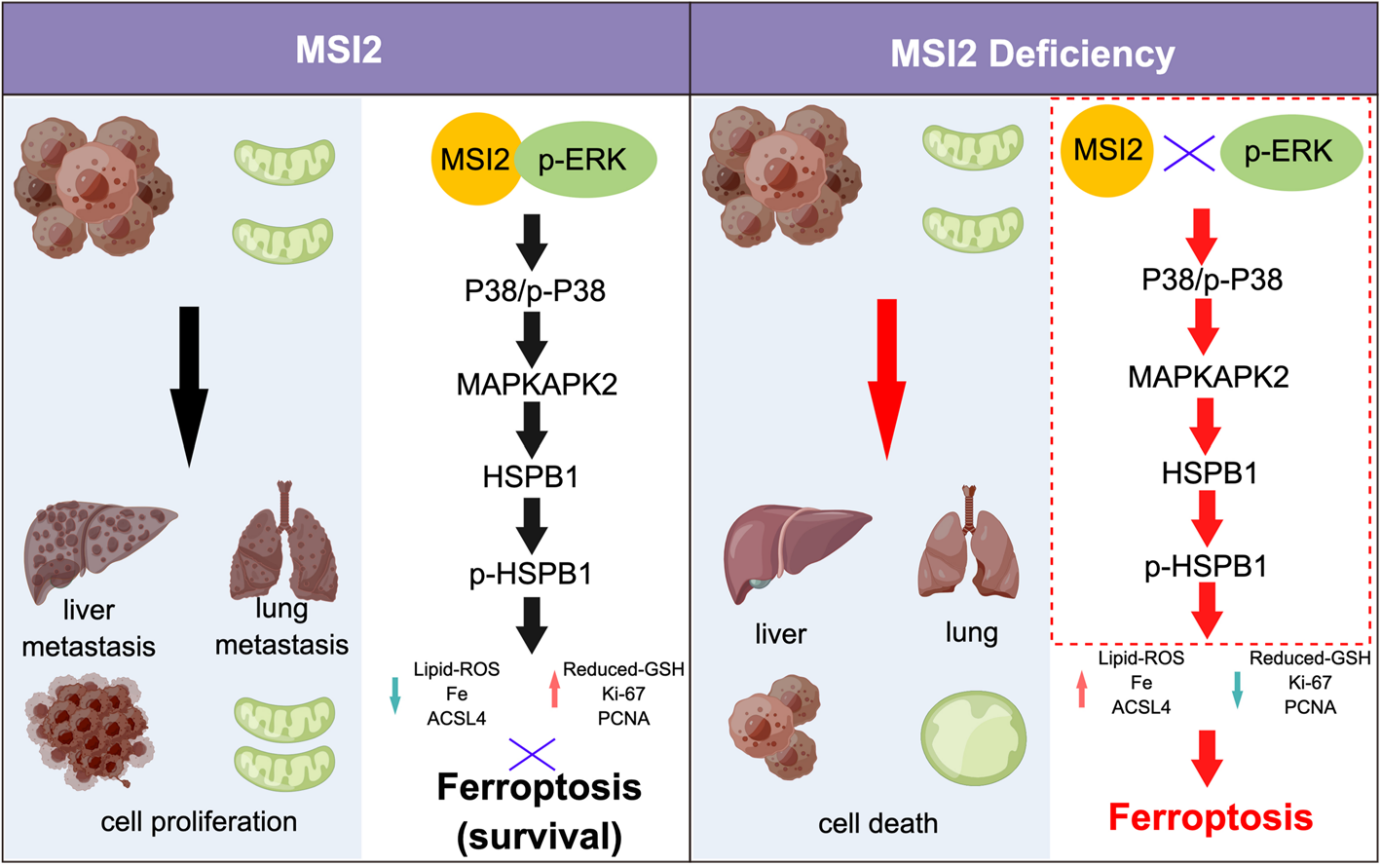
Schematic diagram of the MSI2-MAPK-HSPB1 axis in CRC ferroptosis. MSI2 deficiency suppresses the growth and survival of CRC cells and promote ferroptosis by reducing interaction with p-ERK and inactivating the MAPK signaling pathway to inhibit HSPB1 phosphorylation, which leads to downregulation of PCNA and Ki67 and upregulation of ACSL4 in cancer cells and subsequently induces redox imbalance, iron accumulation and mitochondrial shrinkage, ultimately triggering ferroptosis

Currently, no data have described the effects of MSI2 on ferroptosis. The present study, for the first time, identified a novel pivotal role of MSI2 in inhibiting CRC ferroptosis: it mainly promotes MAPKAPK2-HSPB1 axis phosphorylation through interacting with P-ERK and then activating the p38-MAPK signaling pathway (Fig. 9). Consistent with previous reports [59, 60], we demonstrated that MSI2-mediated HSPB1 expression was required for CRC cell malignancy, and genetic knockdown of MSI2 significantly decreased the growth, proliferation, migration, invasion and metastasis of CRC cells in vitro and in vivo (Figs. 2 and 6). Notably, in addition to involving neurological disorders such as Parkinson’s disease, Huntington’s disease and Alzheimer’s disease [31, 61], and our proteomics analysis also revealed that MSI2 depletion was closely connected to the lipid transport and metabolism process, inorganic ion transport and metabolism process, oxidative phosphorylation and especially the MAPK signaling pathway (Figs. 4 and 5). And MAPK activation is involved in various cellular processes such as cell growth, differentiation, proliferation and development [62]. A previous study also indicated that MSI2 knockdown contributes to the inactivation of the ERK/MAPK and p38/MAPK pathways to regulate cell proliferation and cell death in leukemogenesis [56]. In addition, ferroptosis is an iron-dependent type of cell death characterized by lipid peroxidation, GSH imbalance, and mitochondrial damage, also involves dysregulation of the MAPK signaling pathway [63]. Further investigation of ferroptosis-related features in CRC cells revealed that MSI2 deficiency significantly increased the total ROS and lipid-ROS levels, erastin-induced ferroptosis cell death, iron and ferrous ion concentrations and proportion of shrunken mitochondria while decreasing the reduced GSH levels (Fig. 3). All these data indicate a novel function of MSI2-mediated MAPK signaling pathway activation in the inhibition of ferroptosis cell death in CRC.

HSP family genes have been identified and confirmed to be involved in regulating the ferroptosis process in different disorders and cancers [33]. Further enrichment analysis of genes corresponding to differentially expressed proteins identified by proteomics showed that the ferroptosis suppressor gene HSPB1 was significantly downregulated after MSI2 knockdown. Consistently, previous studies have indicated that HSPB1 negatively regulates intracellular iron uptake and accumulation, and its phosphorylation mediates resistance to ferroptosis [34, 38, 64, 65]. Here, we provide evidence that MSI2 knockdown also inhibits HSPB1 phosphorylation at the Ser-78 site. Interestingly, based on immunoprecipitation analysis, we found that there was no direct interaction between MSI2 and HSPB1, but MSI2 could direct interact with p-ERK and regulate the phosphate levels of downstream cascade genes. In addition, the interactions between MAPK family genes and HSPB1 were evidenced by PPI networks and previous reports [66, 67]. The HSPB1 protein can serve as a downstream substrate for MAPKAPK2 kinase, and MAPKAPK2 activation can further induce HSPB1 phosphorylation in response to stress, thereby effectively preventing oxidative stress injury [46–48]. In this study, we demonstrated that MAPK cascade activation and HSPB1 phosphorylation activated by MSI2 could protect CRC cells from ferroptosis (Figs. 5 and 6). More importantly, restoration of HSPB1 expression rescued the effects of MSI2 deficiency on CRC ferroptosis in vitro and in vivo, further indicating the protective effects of HSPB1 in inhibiting ferroptosis (Figs. 7 and 8). Thus, cancer cells may be more predisposed to ferroptosis cell death if the MAPK-HSPB1 axis signaling pathway is inactivated.

To date, many driver and suppressor genes associated with ferroptosis cell death have been identified in different disorders and cancers [68]. Ferroptosis cell death can be triggered by the upregulation of driving genes and the downregulation of inhibiting genes. In this study, we also evaluated the expression of MSI2, HSPB1 and ferroptosis-related genes from TCGA datasets and proteomics analyses, and it was found that MSI2 and HSPB1 expression were positively correlated with ferroptosis suppressor genes but negatively correlated with ferroptosis suppressor genes. Given the pathogenic roles of MSI2 in tumor progression [21], as well as the protective roles of HSPs in ferroptosis [33–35, 37, 69], we demonstrated that MSI2 could regulate multiple HSPs to repress ferroptosis, especially the ferroptosis suppressor genes HSPB1 and HSPA5, as evidenced by previous studies [13, 34, 39]. Moreover, in clinical CRC specimens, we found that high MSI2 expression indicated higher TUNEL and HSPB1 staining intensities, and MSI2 expression was positively correlated with HSPB1 expression. In addition, the survival analysis based on the transcriptional expression of MSI2 and HSPB1 from the TCGA, GSE17536 and GSE14333 CRC datasets revealed that high MSI2 and HSPB1 expression predicted a poorer prognosis than low expression.

In conclusions, this study elucidated the mechanism by which MSI2 regulates CRC cell ferroptosis for the first time. MSI2 deficiency suppresses the growth and survival of CRC cells to promote ferroptosis through inactivating the MAPK signaling pathway to inhibit HSPB1 phosphorylation, which leads to downregulation of PCNA and Ki67 and upregulation of ACSL4 in cancer cells and subsequently induces redox imbalance, iron accumulation and mitochondrial shrinkage, ultimately triggering ferroptosis (Fig. 9). Therefore, the induction of ferroptosis by inhibition of the MSI2/MAPK/HSPB1 regulatory axis might be a novel therapeutic option for CRC.

### Supplementary Information

**Additional file 1.**

**Additional file 2.**

## Data Availability

The datasets generated and analyzed during the present study are available as Supplementary Information and from the corresponding authors on reasonable request. Online-available datasets were downloaded from GEO (https://www.ncbi.nlm.nih.gov/geo/) and TCGA (https://portal.gdc.cancer.gov/).

## Abbreviations

RBP: RNA-binding protein
CRC: Colorectal cancer
CAC: Colitis-associated colon cancer
TCGA: The Cancer Genome Atlas
GEO: Gene Expression Omnibus
GO: Gene Ontology
KEGG: Kyoto Encyclopedia of Genes and Genomes
COAD: Colon adenocarcinoma
READ: Rectum adenocarcinoma
GSEA: Gene set enrichment analysis
HSPs: Heat shock proteins
CCLE: Cancer Cell Line Encyclopedia
GSH: Glutathione
MMP: Mitochondrial membrane potential
ROS: Reactive oxygen species
MF: Molecular function
qRT‒PCR: Quantitative real-time PCR
IHC: Immunohistochemical
IFC: Immunofluorescence
