# Supplementary material for: Musashi-2 Deficiency Triggers Colorectal Cancer Ferroptosis by Downregulating the MAPK Signaling Cascade to Inhibit HSPB1 Phosphorylation
Source: Biol Proced Online. 2023 Dec 1;25:32. doi: 10.1186/s12575-023-00222-1 (PMC10691036; doi:10.1186/s12575-023-00222-1)
Supplement: Supplementary file 1 — Additional file 1. [file 12575_2023_222_MOESM1_ESM.docx]

Supplementary Materials for

**Musashi-2 deficiency triggers colorectal cancer ferroptosis by downregulating the MAPK signaling cascade to inhibit HSPB1 phosphorylation**

Xiaole Meng^1,2,3,4†^, Xiao Peng^4†^, Wanxin Ouyang^1,4†^, Hui Li^3†^, Risi Na^1,4^, Wenting Zhou^1,4^, Xuting You^1,3^, Yuhuan Li^1,3^, Xin Pu^1,3^, Ke Zhang^1,3^, Junjie Xia^1^, Jie Wang^4^, Guohong Zhuang^1*^, Huamei Tang^1,3*^, Zhihai Peng^1,4*^

**Affiliations**

^1^Organ Transplantation Institute of Xiamen University, Fujian Provincial Key Laboratory of Organ and Tissue Regeneration, School of Medicine, Xiamen University, Xiamen 361102, China

^2^National Institute for Data Science in Health and Medicine, Xiamen University, Xiamen 361102, China

^3^Department of Pathology, Xiang'an Hospital of Xiamen University, School of Medicine, Xiamen University, Xiamen 361102, China

^4^ Department of General Surgery, Organ Transplantation Clinical Medical Center of Xiamen University, Xiang'an Hospital of Xiamen University, School of Medicine, Xiamen University, Xiamen 361102, China

†These authors have contributed equally to this work.

Correspondence to:

Zhihai Peng (zhpeng@xah.xmu.edu.cn);

Guohong Zhuang ([zhgh@xmu.edu.cn](mailto:zhgh@xmu.edu.cn));

Huamei Tang ([tanghuamei2014@163.com](mailto:tanghuamei2014@163.com));

**This file includes:**

Supplementary Figures, S1 to S3

**
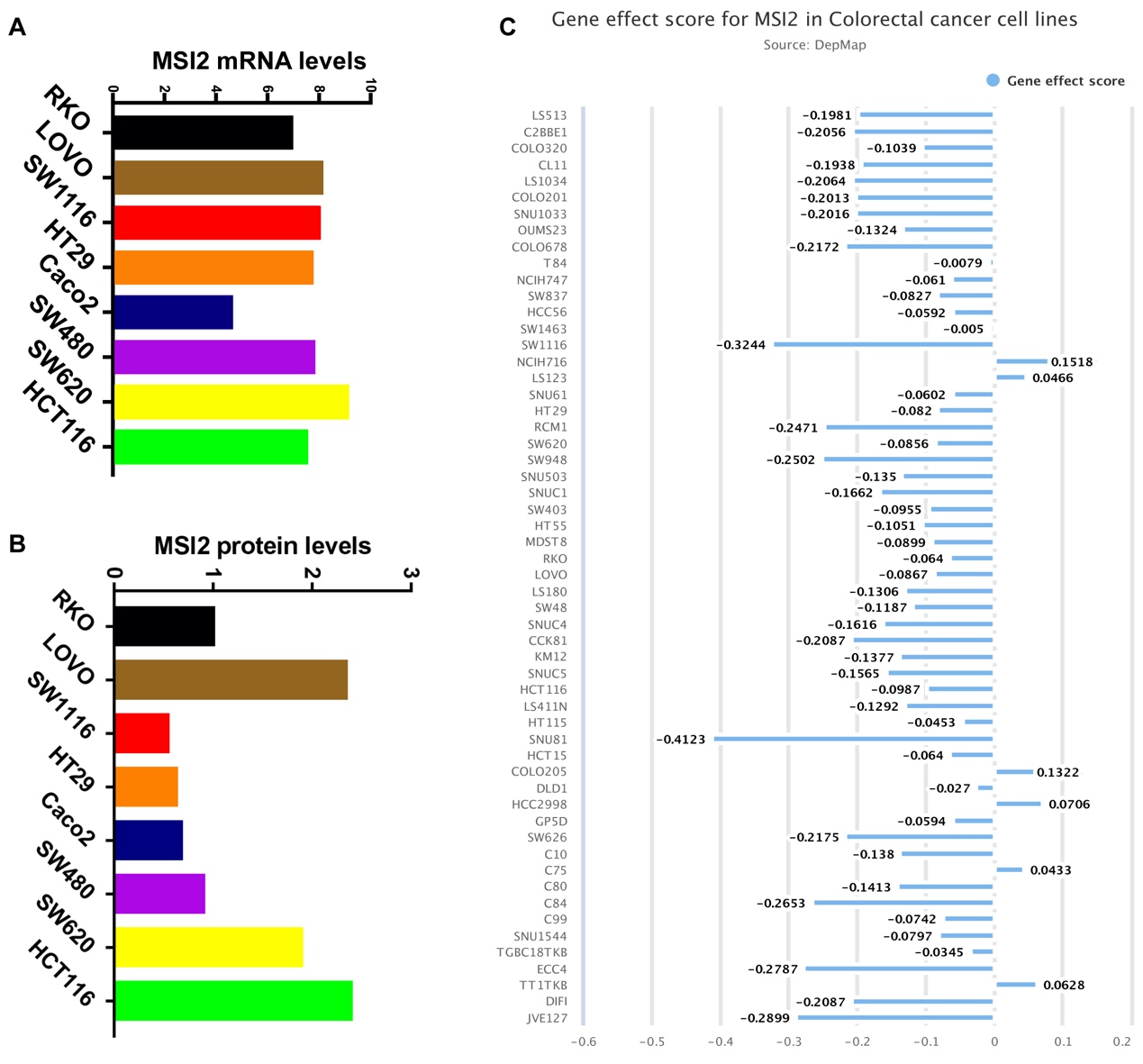
**

**Fig.S1**

**A**, The mRNA expression levels of MSI2 in CRC cell lines. **B**, The protein expression levels of MSI2 in CRC cell lines. **C**, Gene effect scores of MSI2 in CRC cancer cell lines by using DepMap analysis from the CCLE datasets.

**
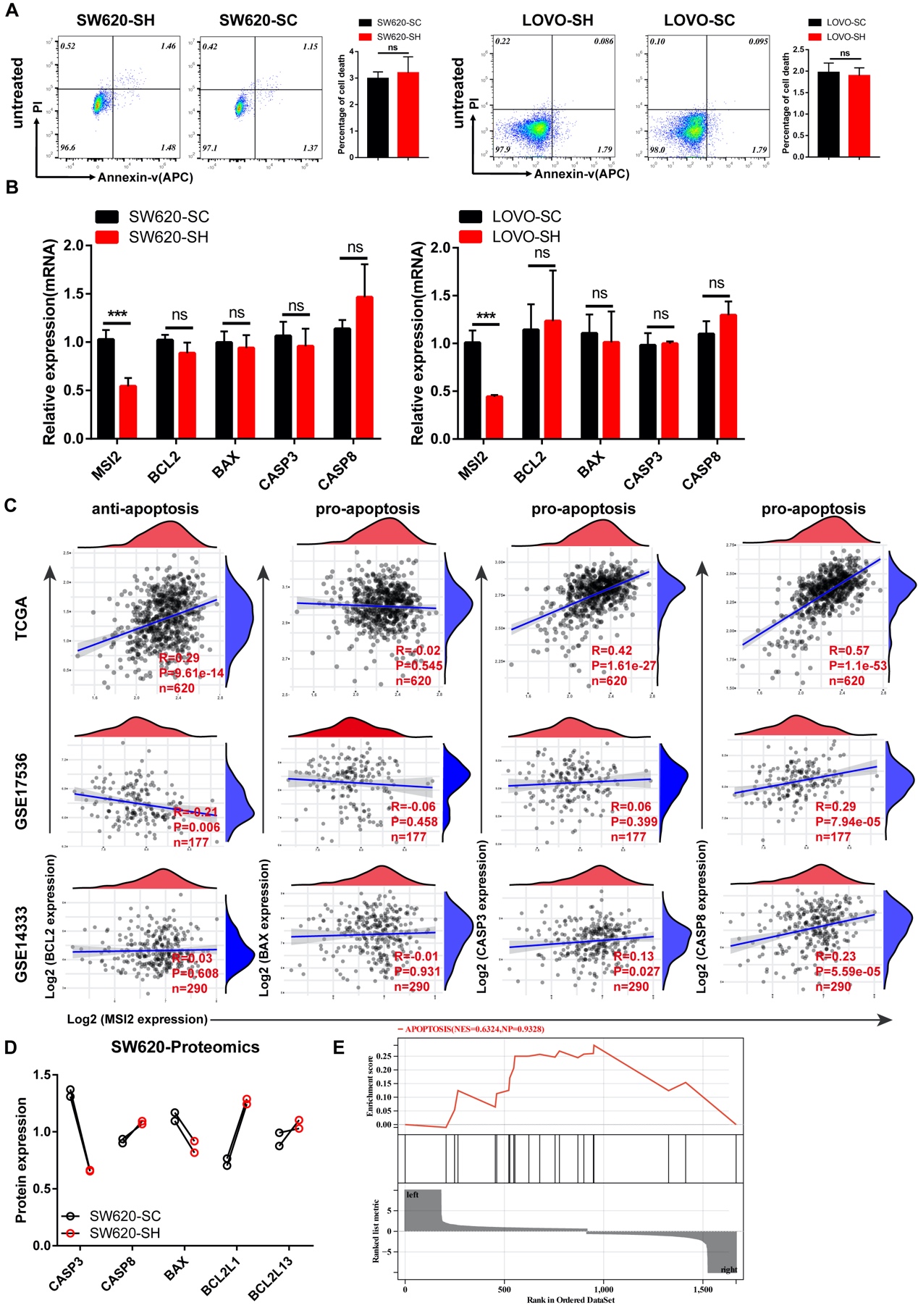
Fig.S2**

**A,** FACS and statistical analysis of cell death rate in SW620 and LOVO stable cells without any treatment. **B**, MSI2 and apoptosis-related genes (BCL2, BAX, CASP3, CASP8) mRNA expression were determined by qRT‒PCR in SW620, LOVO stable cells, the primer sequences are: BCL2: F: GGTGGGGTCATGTGTGTGG, R: CGGTTCAGGTACTCAGTCATCC; BAX: F: CCCGAGAGGTCTTTTTCCGAG, R: CCAGCCCATGATGGTTCTGAT; CASP3: F: GAAATTGTGGAATTGATGCGTGA, R: CTACAACGATCCCCTCTGAAAAA; CASP8: F: AGAGTCTGTGCCCAAATCAAC, R: GCTGCTTCTCTCTTTGCTGAA. **C**, The correlation between MSI2 expression and apoptosis-related gene (BCL2, BAX, CASP3, CASP8) expression was analyzed by *Spearman* correlation analysis from the TCGA (n=620), GSE17536 (n=177) and GSE14333 (n=290) database. **D**, The apoptosis-related gene (CASP3, CASP8, BAX, BCL2L1, BCL2L13) protein expression was analyzed in SW620 Proteomics. **E**, GSEA enrichment analysis in SW620 proteomics showed no significant differences in Apoptosis signaling pathway (NES=0.6324, *p*=0.9328). These results are presented as the mean ± SD values; ns, no significant, ****p*< 0.001; (**A-B**) unpaired 2-tailed Student’s t test.


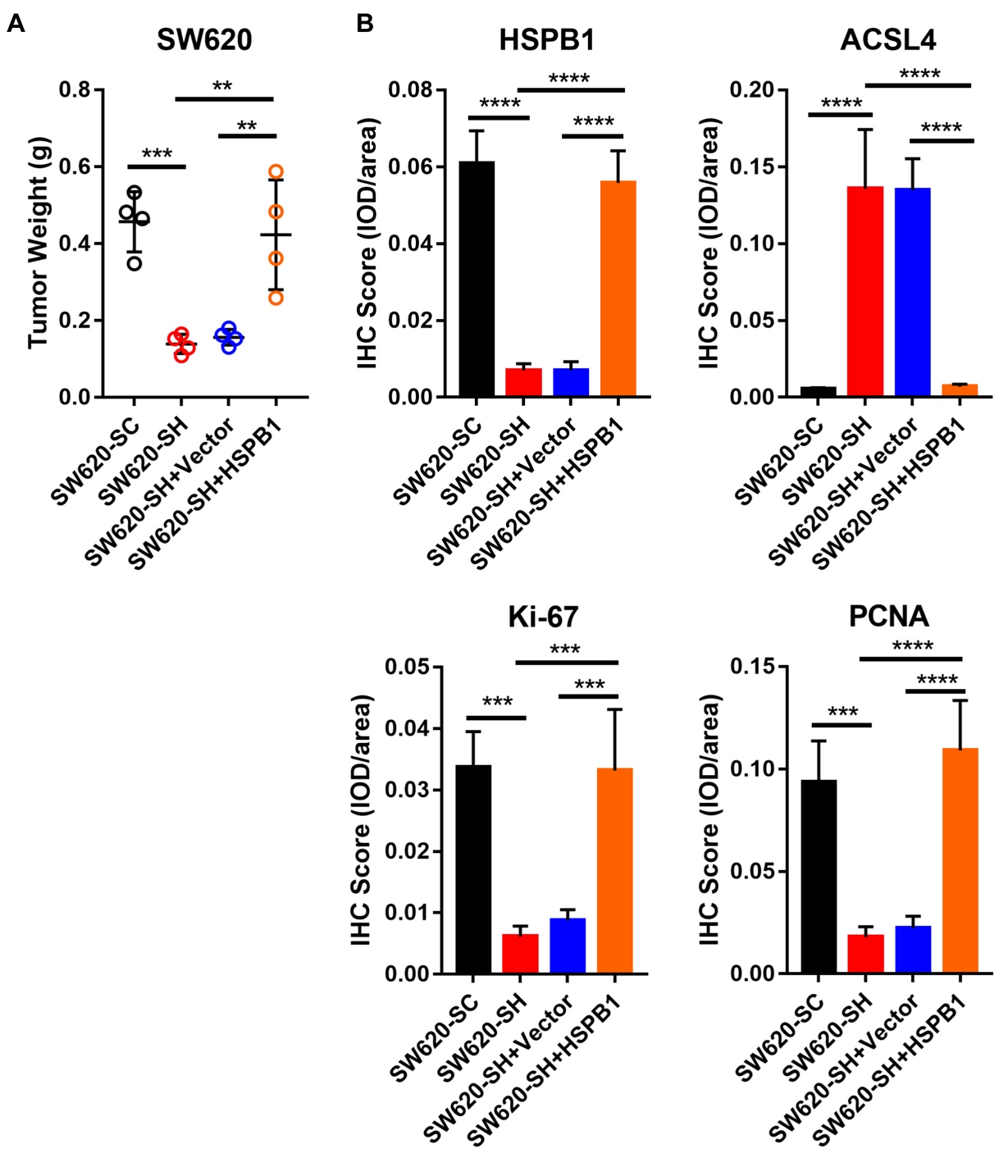


**Fig.S3**

**A**, Statistical analysis of tumor weight in SW620 stable cells rescued with control vector or HSPB1 plasmids in M-NSG mice xenograft tumors, n=4. **B**, Statistical analysis of IHC score (IOD per area) for HSPB1, ACSL4, Ki67 and PCNA from SW620 stable cells rescued with control vector or HSPB1 plasmids in M-NSG mice xenograft tumors. These results are presented as the mean ± SD values; ns, no significant, ***p*< 0.01, ****p*< 0.001, *****p*< 0.0001;(**A-B**) One-way ANOVA test.
